# In silico analyses of *Wnt1* nsSNPs reveal structurally destabilizing variants, altered interactions with Frizzled receptors and its deregulation in tumorigenesis

**DOI:** 10.1038/s41598-022-19299-x

**Published:** 2022-09-02

**Authors:** Amalesh Mondal, Debarati Paul, Shubhra Ghosh Dastidar, Tanima Saha, Achintya Mohan Goswami

**Affiliations:** 1 Department of Physiology, Katwa College, Purba Bardhaman, Katwa, West Bengal 713130 India; 2 Department of Molecular Biology and Biotechnology, University of Kalyani, Nadia, Kalyani, India; 3 Division of Bioinformatics, Bose Institute, P-1/12 CIT Scheme VII M, Kolkata, 700054 India; 4 Department of Physiology, Krishnagar Govt. College, Nadia, Krishnagar, West Bengal 741101 India

**Keywords:** Computational biology and bioinformatics, Molecular biology, Structural biology, Systems biology

## Abstract

Wnt1 is the first mammalian *Wnt* gene, which is discovered as proto-oncogene and in human the gene is located on the chromosome 12q13. Mutations in *Wnt1* are reported to be associated with various cancers and other human diseases. The structural and functional consequences of most of the non-synonymous SNPs (nsSNPs), present in the human *Wnt1* gene, are not known. In the present work, extensive bioinformatics analyses are used to screen 292 nsSNPs of *Wnt1* for predicting pathogenic and harmless polymorphisms. We have identified 10 highly deleterious nsSNPs among which 7 are located within the highly conserved areas. These 10 nsSNPs are also predicted to affect the post-translational modifications of Wnt1. Further, structure based stability analyses of these 10 highly deleterious nsSNPs revealed 8 variants as highly destabilizing. These 8 highly destabilizing variants were shown to have high BC score and high RMSIP score from normal mode analyses. Based on the deformation energies, obtained from the normal mode analyses, variants like G169A, G169S, G331R and G331S were found to be unstable. Molecular Dynamics (MD) simulations revealed structural stability and fluctuation of WT Wnt1 and its prioritized variants. RMSD remained fluctuating mostly between 4 and 5 Å and occasionally between 3.5 and 5.5 Å ranges. RMSF in the CTD region (residues 330–360) of the binding pocket were lower compared to that of WT. Studying the impacts of nsSNPs on the binding interface of Wnt1 and seven Frizzled receptors have predicted substitutions which can stabilize or destabilize the binding interface. We have found that Wnt1 and FZD8-CRD is the best docked complex in our study. MD simulation based analyses of wild type Wnt1-FZD8-CRD complex and the 8 prioritized variants revealed that RMSF was higher in the unstructured regions and RMSD remained fluctuating in the region of 5 Å ± 1 Å. We have also observed differential *Wnt1* gene expression pattern in normal, tumor and metastatic conditions across different tissues. *Wnt1* gene expression was significantly higher in metastatic tissues of lungs, colon and skin; and was significantly lower in metastatic tissues of breast, esophagus and kidney. We have also found that Wnt1 deregulation is associated with survival outcome in patients with gastric and breast cancer. Furthermore, these computationally screened highly deleterious nsSNPs of *Wnt1* can be analyzed in population based genetic studies and may help understand the Wnt1 associated diseases.

## Introduction

The Wnt family consists of secreted glycoprotein signaling molecules which play crucial roles in development and maintenance of many tissues^1^. The name Wnt, came from combination of two genes—*wg* (wingless) and *int-1* (*Wnt1*)^2^. In human, 19 *Wnt* genes were identified with different levels of sequence similarities among the Wnt proteins^3^. Among them, *Wnt1* was the first mammalian Wnt gene, discovered as proto-oncogene and it was capable of driving mammary gland tumor formation^4^. In human, the *Wnt1* gene was located on chromosome 12q13, adjacent to the *Wnt10b* gene^5^. The Wnt1 is essential for oncogenesis and multiple developmental processes like embryonic brain and central nervous system (CNS). Wnt1 mainly activate the canonical Wnt/β-catenin signaling cascade^6^. The transient expression of *Wnt1* induces the formation of β-catenin–LEF1 complex in nucleus, resulting in persistent activation of canonical Wnt/β-catenin signaling^6^. Impaired Wnt signaling due to p.C218G mutation in Wnt1, causes significant and progressive changes in the spine, present both in bony vertebrae and in cartilaginous tissues and become increasingly severe with age over 50 years^7^. Literature studies have also shown that the malfunction of Wnt1 is associated with various cancers, genetic type XV osteogenesis imperfecta, osteoporosis, and neurological diseases^8,9^. High levels of *Wnt1* expression was found in patients with advanced metastasis and the overall survival rate is lower in patients with *Wnt1*-positive cancer^10^. Therefore, the *Wnt1* gene is considerably being focused in the field of human medical genetics^7,10^. After completion of human genome project, many SNPs have been recognized that could be very useful for the study of genotype–phenotype association. The structural and functional consequences for most of the non-synonymous single nucleotide polymorphisms (nsSNPs), present in the human *Wnt1* gene, are still uncharacterized. So, to understand the link between the *Wnt1* genetic information and the phenotype it produces, the identification and characterizations of *Wnt1* genetic variants (especially nsSNPs) are required. Hence, in the present work, the effects of *Wnt1* nsSNPs are investigated for understanding the molecular mechanisms of disease-associated mutationsand discriminating pathogenic and harmless polymorphisms. Being a ligand of Wnt/β-catenin signaling cascade, Wnt1 takes part in a vital protein–protein interaction with Cysteine-rich Wnt-binding domain (CRD) of frizzled receptors (FZD-CRDs). So, in order to understand the effects of disease-related nsSNPs at molecular level; it is also important to consider the impact of nsSNPs on these binding interfaces. Thus, the present study has been carried out using extensive computational tools to extend and explore the impact of nsSNPs on the structure, stability and functional consequences of human Wnt1 with special emphasis on Wnt1-FZD-CRDs interaction.

## Materials and methods

### Retrieval of *Wnt1* nsSNPs

Human *Wnt1* gene SNPs and its protein sequences in the FASTA format were retrieved from the Ensembl genome browser (https://asia.ensembl.org/Homo_sapiens/Gene/Variation_Gene/Table?db=core;g=ENSG00000125084;r=12:48978322-48982620; accessed on 14.10.2020) and UniProt (https://www.uniprot.org/uniprot/P04628) respectively.

### Sequence based screening of deleterious and damaging nsSNPs

The deleterious or damaging nature of nsSNPs in *Wnt1* gene was predicted using multiple sequence based methods like the Sorting Intolerant from Tolerant (SIFT) (http://sift.jcvi.org)^11^, PolyPhen-2 server (http://genetics.bwh.harvard.edu/pph2)^12^, PROVEAN (http://provean.jcvi.org)^13^, Combined Annotation-Dependent Depletion (CADD)^14^, REVEL^15^, Meta Lr^16^, Mutation Assessor (http://mutationassessor.org/r3/)^17^, SNPs&GO^18^, PhD-SNP^19^, PANTHER^20^ and FATHMM^21^, ENTPRISE (http://cssb2.biology.gatech.edu/ENTPRISE/)^22^ tools. The SIFT scores, CADD scores, REVEL scores, MetaLR scores and Mutation Assessor scores for Wnt1 variants were directly obtained from the Ensembl variation table. I-Mutant 3.0 (http://gpcr2.biocomp.unibo.it/cgi/predictors/I-Mutant3.0/I-Mutant3.0.cgi)^23^ a SVM based method, was used to predict the change in protein stability upon mutation by using the sequence of human Wnt1. The value of ΔΔG < 0 was considered as decreased stability of the protein upon amino acid substitution.

### Prediction of physico-chemical properties, secondary structural details and multiple sequence alignment

ExPasy’s ProtParam tool (https://web.expasy.org/protparam/) was used to calculate the physico-chemical properties of Wnt1 protein^24^. Hydropathycity of Wnt1 was predicted using ProtScale (https://web.expasy.org/protscale/) of ExPasy server^25^. Flexibility of the protein was also predicted by the ExPasy server^26^. The PrediSi (PREDIction of SIgnal peptides) (http://www.predisi.de/predisi/index.html) tool was used for predicting Wnt1 signal peptide sequence and its cleavage positions^27^. Secondary structure of Wnt1 was predicted by PSIPRED server (http://bioinf.cs.ucl.ac.uk/psipred/)^28^. GlobPlot 2.3 (http://globplot.embl.de/) was used for predicting the disorder and globular segments Wnt1 protein^29^. Multiple sequence alignment (MSA) of Wnt1 protein sequences were performed using Clustal Omega^30^ (https://www.ebi.ac.uk/Tools/msa/clustalo/).

### Evolutionary conservation and protein–protein interaction analysis

The ConSurf server (http://consurf.tau.ac.il/index_proteins.php) was used to estimates the evolutionary conservation of amino acid positions in the human Wnt1 protein sequence. ConSurf prediction is based on the phylogenetic relations between homologous sequences by using empirical Bayesian inference^31^. Human Wnt1 protein sequence was used as input and the ConSurf was run through ConSeq mode for searching close homologous sequences using CSI-BLAST against the UNIREF-90 protein database. STRING (https://string-db.org/) was used to predict the protein–protein interaction partners of human Wnt1 protein by selecting the *Homo sapiens* in the “Organism” tab of drop down list^32^.

### Prediction of post-translational modifications due to nsSNPs by MutPred2

The impact of nsSNPs on post-translational modification of Wnt1 was studied using MutPred2 (http://mutpred.mutdb.org) to classify amino acid substitutions as pathogenic or benign. A combination of high general scores (g score > 0.75) and low property scores (p score < 0.05) are considered as confident hypotheses^33^.

### Structure modeling, 3D assessment and model refinement

Homology modeling of Wnt1 was carried out to predict its three dimensional (3D) structure as there was no available crystal structure of Wnt1. For homology modeling of Wnt1, web based servers like I-TASSER (https://zhanglab.ccmb.med.umich.edu/I-TASSER/)^34^, Phyre2 (http://www.sbg.bio.ic.ac.uk/~phyre2/html/page.cgi?id=index)^35^, RaptorX (http://raptorx.uchicago.edu/)^36^, Swiss Model (https://swissmodel.expasy.org/interactive)^37^ were used. The predicted models were then validated using ERRAT^38^ and PROCHECK^39^ from SAVES v6.0 (https://saves.mbi.ucla.edu/), PDBsum (http://www.ebi.ac.uk/thornton-srv/databases/pdbsum/Generate.html)^40^, ANOLEA (http://melolab.org/anolea/)^41^ and QMEANDisCo (https://swissmodel.expasy.org/qmean/)^42^. Galaxy Refine (http://galaxy.seoklab.org/cgi-bin/submit.cgi?type=REFINE) was used for structure refinement of the predicted models^43^.

### Stability prediction of Wnt1 upon nsSNPs

The following servers were used for prediction of thermodynamic stability of Wnt1 protein upon amino acid substitutions: mCSM (http://biosig.unimelb.edu.au/mcsm/stability)^44^, SDM (http://marid.bioc.cam.ac.uk/sdm2/)^45^, DUET (http://biosig.unimelb.edu.au/duet/stability)^46^, INPS-3D (https://inpsmd.biocomp.unibo.it/inpsSuite/default/index3D)^47^, and MUpro (http://mupro.proteomics.ics.uci.edu/)^48^. Protein dynamics study provides the link between protein structure and function^49^. Normal mode analysis (NMA) has become a valuable tool for capturing the biologically relevant conformational motions. The intrinsic mobility patterns of both Wnt1 protein and its various pathogenic variants were identified using the WEBnm@ server (http://apps.cbu.uib.no/webnma/home). The comparative analysis mode NMA in WEBnm@ server was used to investigate the proteins dynamic similarity in terms of Bhattacharyya coefficient (BC) and Root Mean Square Inner Product (RMSIP)^50^.

### Estimation of structural deviation in Wnt1 variants

The most deleterious and destabilized variants of Wnt1 were modelled using I-TASSER. The best model was selected on the basis of C-score. Typically, C-score value is in the range of − 5 to 2, where a higher C-score value signifies a model with a high confidence and vice-versa^34^. All the protein structures were then visualized, analyzed in Chimera 1.11^51^.

### Modeling of frizzled-CRD domains and their docking against Wnt1

Wnt ligands bind at the Cysteine-rich Wnt-binding domain (CRD) of frizzled receptors (FZDs). Thus, in the present work, Wnt1 was docked against the CRDs of FZD-1, FZD-2, FZD-4, FZD-5, FZD-7, FZD-8 and FZD-10. The PDB structure of the CRD region of FZD- 4, FZD-5, FZD-7 and FZD-8 were available; and the rest of the FZD-CRDs were modelled. Human FZD-1 CRD was modelled using FZD-7 CRD (PDB ID: 5T44) as a template; FZD-2 CRD and FZD-10 CRD regions were modelled using FZD-7 CRD (PDB ID: 5T44) and FZD-4 CRD (PDB ID: 5BPB) structures as templates, respectively^52^. Molecular docking analyses were carried out using HDOCK (http://hdock.phys.hust.edu.cn/) and the docking results were further validated using HADDOCK 2.4 (https://wenmr.science.uu.nl/haddock2.4/), and ClusPro 2.0 (https://cluspro.bu.edu/publications.php)^53–55^. The interaction 2D plots between Wnt1 and frizzled receptors were analyzed using LigPlot+ (v 2.2.5)^56^ and PDBSum.

### Impact of nsSNPs on the stability of Wnt1-FZD-CRD binding complexes

The effects of 8 most destabilizing and deleterious nsSNPs and other nsSNPs present on the binding interface of Wnt1-FZD-CRD complexes were analyzed by mCSM. mCSM predicts the impact of mutations on the binding affinity of protein–protein complexes in both regression and classification tasks (i.e. prediction of the numerical change or its direction)^44^.

### Molecular dynamic (MD) simulation of WT and mutated Wnt1 in apo and Wnt1-FZD-CRD complexed conditions

To evaluate the stability of the modelled structures, MD Simulation was done and the list of which was provided in Supplementary Table S1. Docking results of HADDOCK 2.4 and ClusPro 2.0 revealed best score with FZD-8 and the structures were almost similar (Supplementary Fig. S1). Wnt1-FZD-8 complex obtained from HADDOCK 2.4 was chosen for simulation optimisation. Each system was solvated using TIP3P model^57^ assuring at least 8 Å thickness of water layer in cubic solvation box, and 0.15 M KCl concentration after neutralization of overall charges appropriately. Following a brief energy minimization, all atom molecular dynamics simulation was run on each system using CHARMM36 force field^58^ implemented through NAMD2.12^59^, under periodic boundary condition. PME^60^ was used to compute long range electrostatic interactions, whereas short range non bonded interactions were truncated at 14 Å with a switching function. Langevin dynamics and Langevin piston methods^61^ were used to maintain NPT condition at 300 K and 1 atm pressure. Time integration step was set to 2 fs after freezing the vibrations of the bonds involving hydrogen using SHAKE algorithm^62^. Heating and equilibration was followed by 20 ns of production MD run for each system. The trajectories were clustered using Chimera 1.11^51^ using default settings. Central frame of the top cluster was selected from each trajectory to model the mutants, i.e. the separate models for A129T, G169A, G169D, G169S, A253S, G312A, G331R, G331S systems, using CHARMM-GUI webserver^63^. Each mutant was simulated using MD for 5 ns, using the same strategies. Total 120 ns simulation was run.

### Differential expression of *Wnt1* gene during cancer formation and its impact on overall survival of cancer patients

We used TNMplot (https://www.tnmplot.com/) to analyse differential expression of *Wnt1* gene from GEO samples in normal (3691 samples), tumor (29,376 samples) and metastatic (453 samples) tissues^64^. Kaplan–Meier plotter (http://kmplot.com/analysis) is a meta-analysis based biomarker prediction tool for the survival assessment of breast, ovarian, gastric and lung cancer patients by integrating gene expression data and clinical data in relation to expression level of genes of interest (http://kmplot.com/analysis)^65^. The probe used for the *Wnt1* gene was “208570_at” and the overall survival analysis was run on 4929 (breast cancer), 1435 (ovarian cancer), 875 (gastric cancer) and 1925 (lung cancer) number of patients. Based on the median value, cancer patients of each type (breast, ovarian, gastric, and lung cancer) were divided into high and low expression groups for comparison and assessment of the cancer patients’ overall survival. Biased arrays were excluded for quality control. The p-values less than 0.05 were considered as statistically significant.

### Statistical analyses

We have used multiple web-servers in our present study and each web-server has its own implemented statistical methods. The statistical significance of differential expression of *Wnt1* between 21 different normal and tumor samples was observed from Mann–Whitney p-value; whereas, in case of 12 different normal, tumor and metastatic samples, Dunn's multiple comparisons test p-value was used. For survival analyses, Kaplan–Meier (log rank) test, the p-value was used to measure the statistical significance.

## Results

### Retrieval and screening of deleterious nsSNPs

In the present study, a total 292 nsSNPs of human *Wnt1* gene were retrieved from the Ensembl (Supplementary Table S2) and were subjected to extensive computational analyses towards predicting their effects on the protein structure, functions and stability. The initial sequence based screening of these nsSNPs were performed using state-of-art computational tools like SIFT, PolyPhen-2, CADD, REVEL, Meta-Lr, Mutation Assessor, PROVEAN, PANTHER, SNPs&GO, PhD-SNP, FATHMM and ENTPRISE (Table 1 and Supplementary Table S3). The SIFT outputs a Tolerance index (TI), which measures functional impacts of amino acid substitution in Wnt1^11^. It was found that out of 292 nsSNPs, 133 nsSNPs were tolerated and 159 nsSNPs (54.45%) were deleterious in nature with the TI score ≤ 0.05. Among these deleterious nsSNPs, 93 nsSNPs had TI score of 0.00; followed by 15, 22, 9, 15, and 5 nsSNPs with TI score of 0.01, 0.02, 0.03, 0.04 and 0.05, respectively. PolyPhen-2 server classifies functionally damaging nsSNPs of *Wnt1* on the basis of PSIC score as “probably damaging” for 63 nsSNPs (PSIC score: 0.449 to 0.908) or “possibly damaging” for 99 nsSNPs (PSIC score: 0.911 to 1). CADD server predicted 77 (26.37%) nsSNPs of *Wnt1* as likely deleterious. Data from REVEL and Meta Lr showed that 131 (44.86%) nsSNPs of *Wnt1* gene had likely disease causing potential and 113 nsSNPs (38.70%) had damaging effects, respectively. According to Mutation Assessor, which predicted the impact of mutation on the function of Wnt1, it was found that 22 nsSNPs had high impact, 90 nsSNPs had medium impact, 81 nsSNPs had low impact and the rests were neutral in nature. PROVEAN classified nsSNPs of *Wnt1* as “Deleterious” (125 nsSNPs; score < − 2.5) or “Neutral” (167 nsSNPs; score > − 2.5) on the basis of alignment-based scoring approach. It was predicted from the SNPs&GO that 97 nsSNPs of *Wnt1* had disease causing potential based on protein sequence, evolutionary information, and function as encoded in the Gene Ontology terms. Another SVMbased classifier PhD-SNP predicted that 142 variants of Wnt1 had disease causing potential. Hidden Markov model based classifier like PANTHER and FATHMM predicted 113 variants as disease causing and 35 variants as damaging respectively. Among these 35 damaging variants of Wnt1 from FATHMM server, 32 variants were also predicted as disease causing by PANTHER. Sequence entropy based prediction from ENTPRISE revealed that 89 variants were deleterious in nature. The selected state-of-the-art tools had covered maximum number of methods (alignment score; neural networks; hidden Markov models; support vector machine; Bayesian classification), used for the prediction of pathogenic nsSNPs. We observed different outputs regarding an amino acid substitution from these twelve tools. Therefore, in this study, a Wnt1 variant was considered as highly damaging/deleterious/pathogenic/disease causing if outputs from all the twelve methods were in agreement^66,67^. Accordingly, initial sequence based screening had revealed 10 nsSNPs of *Wnt1* as highly deleterious (viz. A129T, G169S, G169D, G169A, C218Y, A253S, G312A, G331S, G331R, and W351G). These 10 highly deleterious nsSNPs were further analyzed by sequence based stability prediction tool I-Mutant 3.0 (Supplementary Table S4), and found that the stability was decreased for all variants except C218Y.

**Table 1:** Summary of initial sequence based screening of 292 nsSNPs by twelve sequence based prediction tools.

Prediction output	Number of nsSNPs present in each prediction tool											
	SIFT	PP-2	CADD	REVEL	ML	MA	PROVEAN	PANTHER	SNP&GO	Ph-D SNP	FATHMM	ENTPRISE
Tolerated	133	–	–	–	179	–	–	–	–	–	257	–
Deleterious	159	–	–	–	–	–	125	–	–	–	–	89
Benign	–	130	–	–	–	–	–	–	–	–	–	–
PSD	–	99	–	–	–	–	–	–	–	–	–	–
PD	–	63	–	–	–	–	–	–	–	–	–	–
LB	–	–	215	161	–	–	–	–	–	–	–	–
LD	–	–	77	–	–	–	–	–	–	–	–	–
LDC	–	–	–	131	–	–	–	–	–	–	–	–
Damaging	–	–	–	–	113	–	–	–	–	–	35	–
Neutral	–	–	–	–	–	99	167	150	195	150	–	203
Low impact	–	–	–	–	–	81	–	–	–	–	–	–
Medium impact	–	–	–	–	–	90	–	–	–	–	–	–
High impact	–	–	–	–	–	22	–	–	–	–	–	–
–	–	–	–	–	–	–	–	113	97	142	–	–
UC	–	–	–	–	–	–	–	29	–	–	–	–

Number of nsSNPs shown for each category.

*PSD* possibly damaging, *PD* probably damaging, *LB* likely benign, *LD* likely deleterious, *LDC* likely disease causing, *UC* unclassified, *PP-2* PolyPhen-2, *ML* meta Lr, *MA* mutation assessor.

### Secondary structure, evolutionary conservation profile and protein–protein interactions of Wnt1 in relation to its nsSNP distribution

Physico-chemical analysis of Wnt1 by ProtParam revealed its theoretical pI of 9.28, aliphatic index of 71.46, theoretical extinction coefficient (at 280 nm) of 62,335 M^−1^ cm^−1^ and the GRAVY score of − 0.347. The average flexibility of Wnt1 was predicted from its sequence by Expasy and found several highly flexible regions. It was also found from GlobPlot analysis (Supplementary Fig. S2) that the protein had six disordered regions (1–5, 9–39, 142–156, 192–217, 317–333 and 339–368) and two potential globular domains (40–141 and 218–316). It was observed that 106 nsSNP sites were present in the predicted globular domains and 65 nsSNP sites were present in the disordered regions of Wnt1. It was evident from the GlobPlot analyses that the 10 highly deleterious variants had no major impact on the disordered regions and globular domains of Wnt1. Wnt1 has a signal sequence in the N-terminal region from 1 to 27 residues and we have found 23 nsSNPs are located in this region. The secondary structural details of Wnt1 showed that random coil (42%) contributed the major portion of the protein, followed by alpha helix (35%), extended strand (16%) and beta turn (7%) regions. The secondary structures of Wnt1 and its 10 highly deleterious variants are shown in Supplementary Fig. S3. It was also observed that out of 10 highly deleterious nsSNPs, only A129T variant was located in alpha-helix region, 7 variants (G169A, G169D, G169S, A253S, G312A, G331R and G331S) in loop region while the rest 2 variants (C218Y and W351G) were located within the extended strand in beta-ladder region. It was also evident from the Supplementary Fig. S3, that these 10 highly damaging nsSNPs had no impact on the secondary structure of Wnt1. The multiple sequence alignment of Wnt1 and its 10 highly deleterious variants is shown in Supplementary Fig. S4.

**Figure 1:**
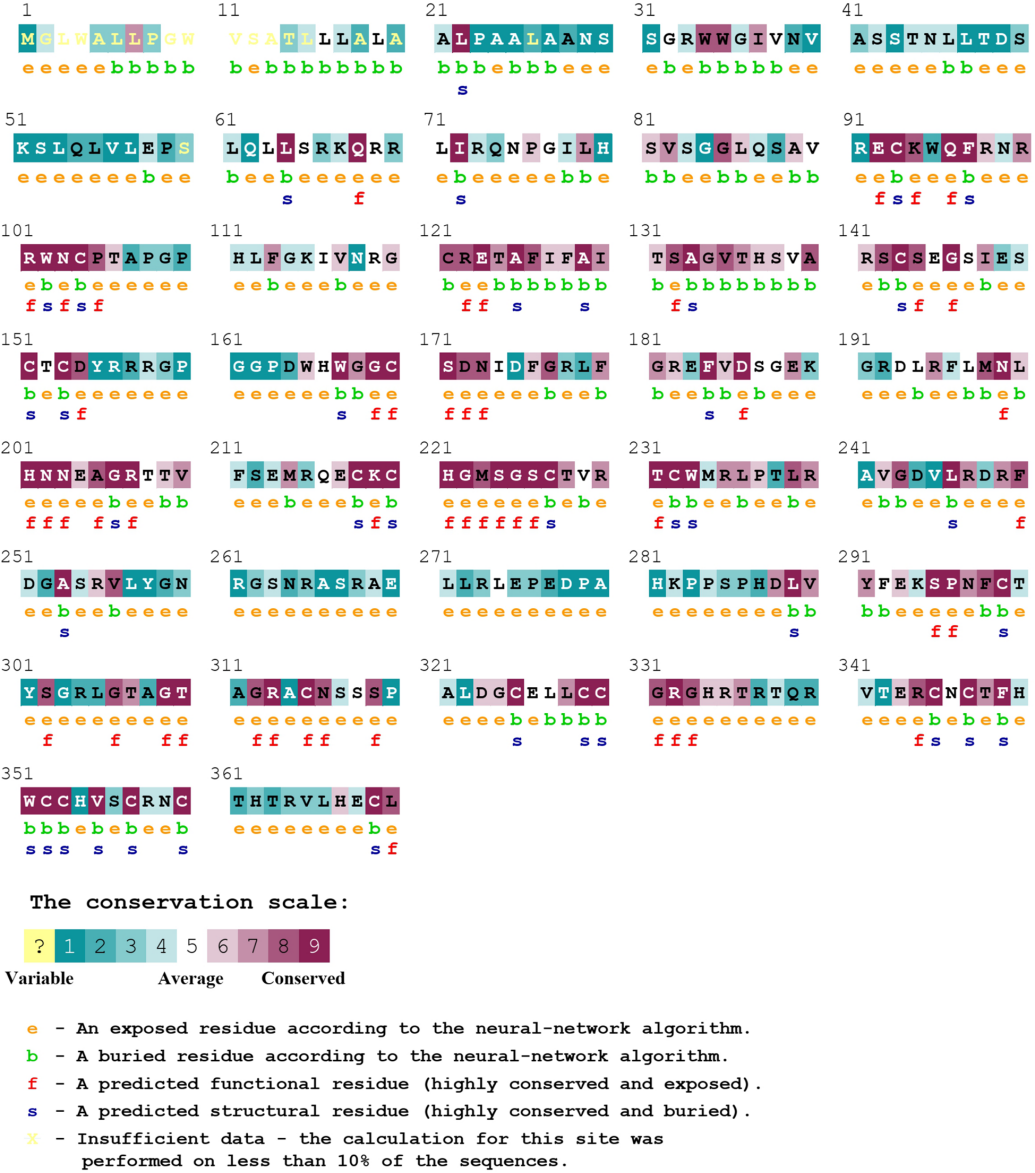
Evolutionary conservation profile of human Wnt1 by ConSurf. The color coding bar shows the coloring scheme representation of conservation score.

The Sequence based evolutionary conservation of human Wnt1 was carried out using phylogenetic relations between homologous sequences by ConSurf. The rate of evolution at each residue was calculated by ConSurf, using the empirical Bayesian method with the best fit method of substitution for proteins. In ConSurf, Multiple Sequence Alignment was built using MAFFT and the homologues were collected from UNIREF90 database with HMMER homology search algorithm (E-value: 0.0001). ConSurf analyses had identified the conserved residues in human Wnt1 protein and predicted the residues to be exposed or buried in the protein structure (Fig. 1). The colour based conservation score (grade 1–9) indicates the evolutionary conservation of a particular position, where 1 indicates rapidly evolving sites and 9 indicates slowly evolving (i.e. evolutionarily conserved) sites. Position wise conservation scores of human Wnt1 protein is given in Supplementary Table S5. It was found that the residues 92–106, 120–144, 198–210 and 217–240 of human Wnt1 protein were highly conserved, whereas the N-terminal and C-terminal regions were highly variable. The 292 variants occurred within the 195 residues of the Wnt1 protein and out of which 61 positions were highly conserved (conservation score: 7 to 9). It was also observed that out of 10 highly deleterious nsSNPs (obtained after initial sequenced based screening), 7 nsSNPs (viz. A129T, G169S. G169D, G169A, C218Y, A253S and W351G) were located in highly conserved areas of protein having conservation score of 9; and rest of the 3 nsSNPs (G312A, G331S, G331R) occurred in residue positions having conservation score of 8.

### Protein–protein interaction prediction by STRING

Determination of protein–protein interaction (PPI) network is important to understand the functional impact of the protein and its interacting partners. It was found that Wnt1 had high confidence score for interaction with CTNNB1, WLS, DVL-1, PORCN, FZD1, FZD8, LRP5, LRP6, RYK and SFRP1 proteins (Supplementary Fig. S5). This decoding of PPI network at cellular level helps further understand the mechanism of the target protein and the possible changes in interaction affinity upon amino acid alteration in Wnt1.

### Predicting the impact of nsSNPs on post-translational modifications (PTM) in Wnt1

Further studies were carried out to assess the impact of 10 highly deleterious nsSNPs on post-translational modifications of Wnt1 by MutPred2 (Supplementary Table S6). Probability scores above threshold (0.5) is considered as ‘harmful’; whereas scores greater than 0.75 signify ‘harmful’ prediction with high confidence^33^. All the 10 highly deleterious nsSNPs were predicted to have ‘harmful’ effect (scores > 0.75) with altered posttranslational modifications and structural features. Amino acid substitutions like G169A, G169D and G169S cause loss of catalytic site at W167 position and gain of disulfide linkage at residue C170. There is a gain of new allosteric site at W167 due to G169A and G169S; but the variant G169D causes loss of that allosteric site. Catalytic site is also lost at H221 due to amino acid substitution C218Y and at residue position C330 due to variant G331S and G331R. Some PTM like residue Y258 acquire new phosphorylation site and residue N346 gain new N-linked glycosylation site due to the amino acid substitution of A253S and W351G respectively. But N-linked glycosylation site at residue N316 is lost due to another amino acid variation of G312A which also introduce a catalytic site at R313 and a disulfide linkage at residue C315. Variant G331S alters the protein stability by acquisition of new beta strand and formation of new disulfide linkage at residue C330 while the same disulfide linkage is lost due to amino acid substitution of G331R. Variant W351G alters the stability of Wnt1 by loss of beta strand and acquisition of intrinsic disorder.

### The impact of nsSNPs on the Wnt1 structure and stability

The functions and ability to interact with ligands depend upon the tertiary structure of the protein^68^. As there was no available crystal structure of Wnt1 in the protein data bank, the structure of Wnt1 was modelled by comparative homology based approach using I-TASSER, Phyre2, RaptorX and Swiss Model (Supplementary Fig. S6). The best structural model was selected based on structural quality assessment and structure validation of these predicted models. The Ramachandran plots of all four predicted models of Wnt1 are also given in Supplementary Fig. S6. Therefore, on the basis of comparative homology modeling and assessment, we used Wnt1 structure, modelled by I-TASSER for further studies. Sequence based screening approach revealed 10 nsSNPs as highly deleterious. These 10 nsSNPs of Wnt1 were then analyzed using mCSM, SDM, DUET, INPS-3D and MUpro to assess the structural stability of the protein upon amino acid substitution (Table 2). The mCSM server predicted all 10 variants as destabilizing (ΔΔG: − 0.248 to − 1.962 kcal/mol). The prediction from SDM server showed that stability were reduced for 8 variants (ΔΔG: − 1.97 to − 4.11 kcal/mol) and increased for 2 variants (ΔΔG: 0.22–0.23 kcal/mol). The rest 3 prediction tools—DUET, INPS-3D and MUpro predicted that these 10 most deleterious variants were destabilizing. By concordance, 8 out of 10 nsSNPs of Wnt1 were found to have structural destabilizing effects (as predicted by all the five stability prediction algorithms). These 10 structural variants of Wnt1 were then modelled using I-TASSER (Supplementary Fig. S7) and the best model for each variant was selected on the basis of their corresponding C-score value. Further analyses of these 10 mutant models along with the wild type Wnt1 in Chimera 1.11 revealed that the mutant residues had different non-covalent bonding interactions than the wild type residues. The structural alterations of these 10 Wnt1 variants were measured by their corresponding RMSD values (Table 3). It was found that the variant G169S had the highest RMSD value of 1.182 Å, while the G331R variant had the lowest RMSD value of 1.008 Å. The coarse grained elastic network model (ENM)-based normal mode analysis (NMA) revealed some important dynamic features of the wild type and mutant Wnt1 (Supplementary Fig. S8). It was found that all the BC values of wild type Wnt1 and its 8 high risk variants were very high and the score was 0.97. A similar picture was also presented by high Root Mean Square Inner Product (RMSIP) value of 0.96. Based on the deformation energies obtained from the normal modes, it was observed that G169A, G169S, G331R and G331S variants were largely unstable.

**Table 2:** Stability prediction of 10 prioritized nsSNPs of Wnt1. Stability was predicted as ΔΔG = ΔG (New Protein) − ΔG (Wild Type) in Kcal/mol.

Variant	mCSM		SDM		DUET		INPS 3D		MUPro	
	DDG (Kcal/Mole)	Outcome	DDG (Kcal/Mole)	Outcome	DDG (Kcal/Mole)	Outcome	DDG (Kcal/Mole)	Outcome	DDG (Kcal/Mole)	Outcome
A129T	− 1.962	Destabilizing	− 2.82	Reduced stability	− 2.272	Destabilizing	− 1.37814	Destabilizing	− 0.83301536	Decrease
G169S	− 1.424	Destabilizing	− 3.56	Reduced stability	− 1.75	Destabilizing	− 0.992932	Destabilizing	− 1.0259415	Decrease
G169D	− 2.346	Destabilizing	− 3.61	Reduced stability	− 2.732	Destabilizing	− 1.0635265	Destabilizing	− 0.84245223	Decrease
G169A	− 0.703	Destabilizing	− 2.81	Reduced stability	− 0.842	Destabilizing	− 1.146045	Destabilizing	− 1.0280278	Decrease
C218Y	− 0.918	Destabilizing	0.22	Increased stability	− 0.539	Destabilizing	− 1.738135	Destabilizing	− 1.4352999	Decrease
A253S	− 1.573	Destabilizing	− 1.97	Reduced stability	− 1.889	Destabilizing	− 1.030433	Destabilizing	− 1.011084	Decrease
G312A	− 0.324	Destabilizing	− 3.56	Reduced stability	− 0.848	Destabilizing	− 0.9703265	Destabilizing	− 0.24808681	Decrease
G331S	− 0.248	Destabilizing	− 4.11	Reduced stability	− 0.834	Destabilizing	− 0.975777	Destabilizing	− 0.89801531	Decrease
G331R	− 0.333	Destabilizing	− 2.26	Reduced stability	− 0.551	Destabilizing	− 0.784194	Destabilizing	− 0.68659603	Decrease
W351G	− 0.506	Destabilizing	0.23	Increased stability	− 0.312	Destabilizing	− 1.23720835	Destabilizing	− 1.7669389	Decrease

**Table 3:** RMSD values of 10 most deleterious Wnt1 variants. The RMSD was measured in Chimera 1.11 using wild type Wnt1 as reference.

Sl. No.	Variant rsID	Wnt1 variants	RMSD (Å)
1	rs764283737	A129T	1.126
2	rs371672410	G169A	1.18
3	rs371672410	G169D	1.09
4	rs773630541	G169S	1.182
5	rs1169010600	C218Y	1.156
6	rs772848874	A253S	1.034
7	rs1442343941	G312A	1.036
9	rs1200558724	G331R	1.008
8	rs1200558724	G331S	1.175
10	rs1057524201	W351G	1.157

**Figure 2:**
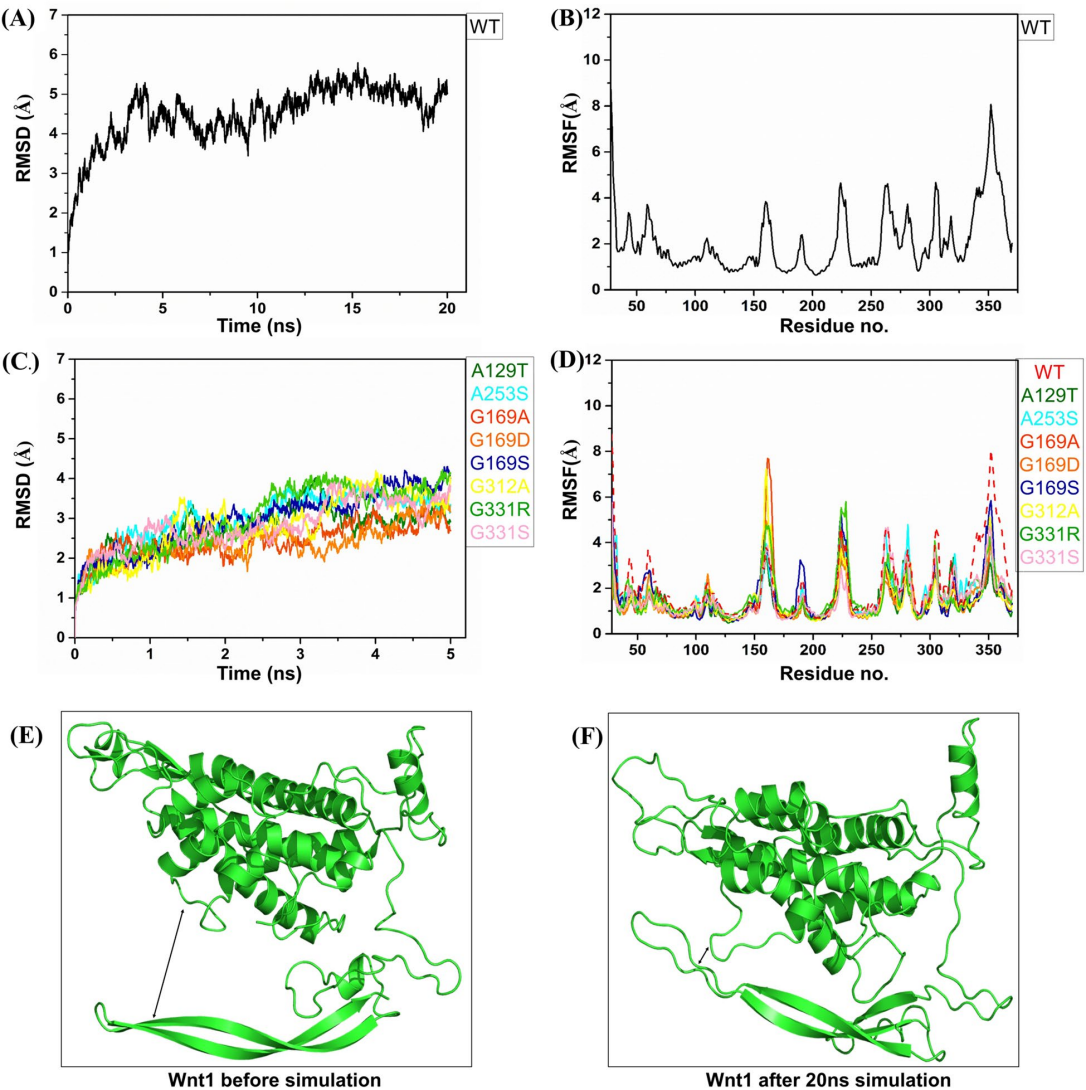
RMSD of Cα atoms of Wnt1 in (**A**) WT and (**C**) Mutated form in apo states. RMSF of Cα atoms of Wnt1 in (**B**) WT and (**D**) Mutated form in apo states. (**E**) Modelled structure of Wnt1 before MD simulation, showing open binding pocket marked in black arrow. (**F**) Structure of Wnt1 after the 20 ns MD simulation, showing the binding pocket is closed (shown in black arrow).

**Figure 3:**
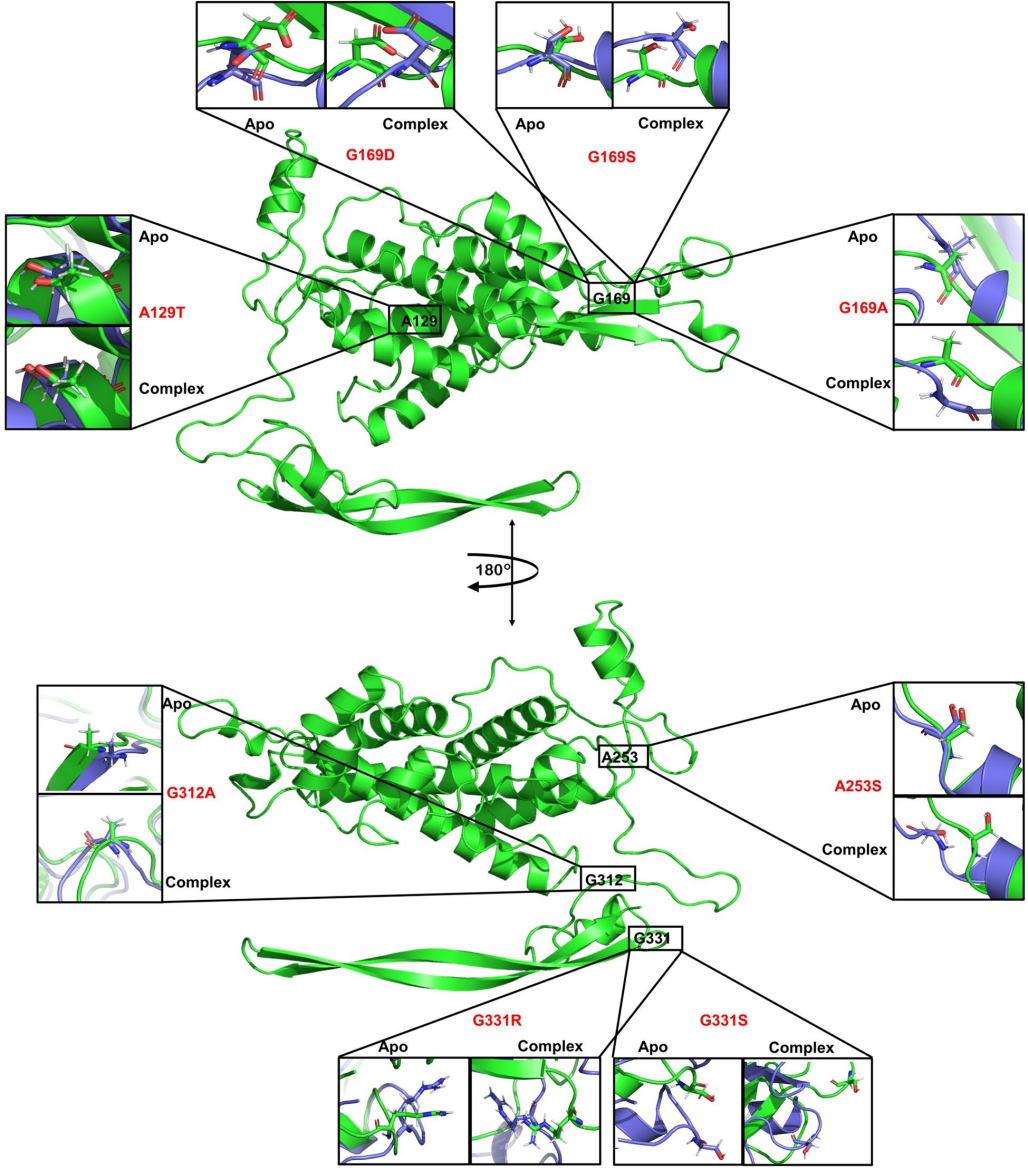
Positions of some selected residues on Wnt1 (full structures, shown in green) have been shown; two orientations have been created with a 180º rotation around an axis that was chosen for the convenience of presentation. The positions of the selected residues have been zoomed in and then the orientations of their side chains of the corresponding mutants in apo and complexes have been shown. The structures before (green) and after (violet) the MD simulation have been shown. Each mutant refers to two zoomed in boxes, i.e. for apo and complex, which have been indicated for each set.

Molecular dynamics (MD) simulations allowed the structural model to relax and to optimize themselves by adjusting internal interactions. Root Mean Square Deviation (RMSD) of the Cα atoms of all apo Wnt1 were computed and plotted as a function of time, taking the initial model as reference. Result of the WT Wnt1 exhibited increment upto 5 ns, which regime reflected the process of optimization of the conformation which was stabilized thereafter and then RMSD remained fluctuating mostly between 4 and 5 Å and occasionally between 3.5 and 5.5 Å (Fig. 2A). The initial rise of RMSD upto ~ 5 Å was because of the event of closure of the binding pocket (Fig. 2E,F) and this observation was in agreement of earlier reports in absence of the ligands^52^. So the MD simulation seemed to be able to benchmark the literature data and reflects the reliability. Root Mean Square Fluctuation (RMSF), of Cα atoms, averaged over the MD trajectories, showed ~ 2 Å fluctuation of the folded region while little higher values were noted in the unstructured loop regions (Fig. 2B). For the mutated systems, the RMSD values were limited within 4.5 Å (Fig. 2C) and the RMSF values followed the trend of < 2 Å for folded regions and higher values for unstructured regions (Fig. 2D). Interestingly, RMSF of the residues within 330–360 (CTD region) were lower compared to that of WT (Fig. 2D). This was because the binding pocket of all the mutants were already closed at the starting point of the MD as they were modelled based on the last conformation obtained after the 20 ns MD run of WT; as mentioned earlier the WT experienced the closure of the binding pocket during MD. Orientation of the side chains of the mutated residues were visually inspected using VMD^69^ before and after the MD simulations; the observed deviations were summarized in Fig. 3.

### Interaction of WNT-1 with frizzled receptors

As Wnt ligands bind at the Cysteine-rich Wnt-binding domain (CRD) of frizzled receptors (FZDs), Wnt1 has been docked against the CRDs of FZD-1, FZD-2, FZD-4, FZD-5, FZD-7, FZD-8 and FZD-10 through HDOCK, HADDOCK and ClusPro server^52^. The N-terminal 27 amino acid long signal sequence of Wnt1 was cleaved off before docking^70^. The interaction interfaces between wild type Wnt1 and FZD-CRDs were then evaluated on the basis of docking score (DS) from HDOCK, Z-score from HADDOCK and balanced weighted score from ClusPro (Table 4). Different types of binding interaction pattern of each docked wild type Wnt1 against seven different frizzled receptors are obtained from PDBsum and the details are given in Supplementary Table S7 and Supplementary Fig. S9. It was observed that Wnt1 had maximum number of total interactions against FZD5-CRD (235 interactions) when docked by HDOCK, FZD4-CRD (172 interactions) when docked by HADDOCK and FZD8-CRD (273 interactions) when docked by ClusPro. Among the seven Wnt1-FZD-CRD complexes, best docking was observed between Wnt1 and FZD8-CRD on the basis of lowest balanced weighted score from ClusPro (− 1068.5) and lowest Z score from HADDOCK (− 2.7). Analyses of interacting interfaces of Wnt1-FZD-CRD complexes revealed that the residue positions of 8 high risk variants were not involved in the binding interfaces. Therefore, we had studied the distal effects of these 8 high risk Wnt1 variants in their interaction with seven FZD-CRDs using HDOCK, HADDOCK and ClusPro and the results were also summarized in Table 4. The docking results were then evaluated for interactions between wild type Wnt1 and FZD-CRDs; and it was observed that most of these variants interfered with the Wnt1-FZD-CRD complex formation. The interaction 2D plots between wild type Wnt1 and seven FZD-CRDs (Supplementary Fig. S9) also revealed the interaction patterns between them.

**Table 4:** Docking results of Wnt1 and its 8 destabilizing variants with CRD region of seven Frizzled receptors. Docking was performed by HDOCK, HADDOCK and ClusPro servers.

FZD-CRDs	Docking servers	Parameters	Docking with Wnt1	Docking with Wnt1 variants							
				A129T	A253S	G169A	G169D	G169S	G312A	G331R	G331S
FZD1-CRD	HDOCK	Docking Score	− 242.82	− 269.25	− 261.64	− 273.85	− 279.32	− 328.2	− 264.95	− 268.64	− 282.8
	HADDOCK	Z-score	− 1.4	0.3	− 2.2	− 1.8	− 2.5	− 1.5	− 2.4	− 1.2	− 1.4
	ClusPro	Balanced Weighted Score	− 929.7	− 1067.3	− 895.2	− 1095.1	− 907.5	− 1139.7	− 883.6	− 1188	− 1060.3
FZD2-CRD	HDOCK	Docking Score	− 280.51	− 295.23	− 277.48	− 266.5	− 311.08	− 269.81	− 270.04	− 267.85	− 279.97
	HADDOCK	Z-Score	− 1.5	− 0.9	− 1.9	− 0.1	− 1.8	− 1.5	− 1.5	− 0.4	− 1.6
	ClusPro	Balanced Weighted Score	− 1006.9	− 1151.8	− 984.5	− 1231.3	− 916.7	− 1184.4	− 938.1	− 1175.3	− 1194.1
FZD4-CRD	HDOCK	Docking Score	− 257.21	− 303.7	− 257.7	− 273.03	− 265.37	− 265.36	− 251.01	− 245.07	− 263.87
	HADDOCK	Z-Score	− 1.8	− 1.9	− 1.8	− 2	− 1.3	− 1.3	− 1.7	− 2.3	− 2.2
	ClusPro	Balanced Weighted Score	− 1037.2	− 1126.3	− 921.5	− 1136.7	− 935.2	− 1250.1	− 868.7	− 990.2	− 1158.9
FZD5-CRD	HDOCK	Docking Score	− 273.88	− 294.74	− 259.32	− 294.19	− 256.31	− 279.18	− 262.6	− 259.26	− 295.06
	HADDOCK	Z-Score	− 1.5	− 1.7	− 2.4	− 1.1	− 1.5	− 1	− 2.2	− 2.2	− 1.4
	ClusPro	Balanced Weighted Score	− 1081.6	− 1156.7	− 1087.8	− 1270.5	− 1058.2	− 1144	− 992.2	− 1205.4	− 1082.7
FZD7-CRD	HDOCK	Docking Score	− 285.71	− 283.38	− 249.74	− 294.31	− 302.7	− 272.45	− 258.4	− 271.22	− 272.45
	HADDOCK	Z-Score	− 2	− 1.6	− 1.9	− 2.4	− 1.2	− 1.3	− 0.9	− 1.6	− 1.3
	ClusPro	Balanced Weighted Score	− 1004.1	− 1192.9	− 951.6	− 1199.6	− 958.7	− 1183.8	− 948.8	− 1198.3	− 1185.2
FZD8-CRD	HDOCK	Docking Score	− 257.45	− 289.88	− 307.06	− 307.06	− 284.95	− 278.17	− 254.14	− 279.09	− 302.31
	HADDOCK	Z-Score	− 2.7	− 0.1	− 0.2	− 1.4	− 1.7	− 0.8	− 1.1	− 2.1	− 1.4
	ClusPro	Balanced Weighted Score	− 1068.5	− 1198.6	− 998.4	− 1137.8	− 980.5	− 1309.1	− 973.1	− 1129.2	− 1121.5
FZD10-CRD	HDOCK	Docking Score	− 282.91	− 288.46	− 256.68	− 267.08	− 250.01	− 273.07	− 268.96	− 282.39	− 282.9
	HADDOCK	Z-Score	− 2	− 0.6	− 1.3	− 2.3	− 1.6	− 1.8	− 1.4	− 2	− 2.2
	ClusPro	Balanced Weighted Score	− 1007.2	− 1189.4	− 910.8	− 1027.1	− 952.1	− 987.7	− 1020.7	− 1083.5	− 1062.3

Further stability based analyses of binding interfaces between Wnt1 variants and FZD-CRDs in mCSM revealed that almost all the highly deleterious variants had destabilizing effects on respective Wnt1- FZD-CRD binding interfaces (Table 5). For this study, we used Wnt1-FZD-CRD complexes, obtained from the three docking servers viz. HDOCK, HADDOCK and ClusPro. It was found from Table 5 that the amino acid substitution A129T and A253S in Wnt1 increased its binding affinity for all the 7 FZD-CRDs; whereas other five substitutions were predicted to decrease the binding affinity for FZD-CRDs. As the 8 highly destabilizing variants of Wnt1 were not present in the binding interfaces with FZD-CRDs, we had included all the interacting residues (Supplementary Fig. S10) of Wnt1 where nsSNPs were reported to investigate their effects on the stability of Wnt1- FZD-CRD complexes (Supplementary Table S8). It was found that most of the amino acid substitutions present in the interaction interface of Wnt1 showed destabilizing or highly destabilizing effects.

**Table 5:** Impact of 8 highly destabilizing variants on the binding interface of Wnt1-FZD-CRDs complex as predicted by mCSM server. Wnt1-FZD-CRDs complexes were obtained from HADOCK, HADDOCK and ClusPro server. ΔΔG (kcal/mol) < 0 indicates decreased binding affinity.

Wnt1 variants	FZD1-CRD			FZD2-CRD			FZD4-CRD			FZD5-CRD			FZD7-CRD			FZD8-CRD			FZD10-CRD		
	Predicted ΔΔG			Predicted ΔΔG			Predicted ΔΔG			Predicted ΔΔG			Predicted ΔΔG			Predicted ΔΔG			Predicted ΔΔG		
	HK	HDK	CP	HK	HDK	CP	HK	HDK	CP	HK	HDK	CP	HK	HDK	CP	HK	HDK	CP	HK	HDK	CP
A129T	0.376	0.099	0.42	0.183	0.192	0.385	0.273	0.108	0.455	0.398	0.182	0.344	0.221	0.05	0.291	0.301	0.186	0.326	0.308	0.143	0.427
G169A	− 0.148	− 0.295	− 1.758	− 0.411	− 0.507	− 0.61	− 0.375	− 0.433	− 1.887	− 1.659	− 0.547	− 0.579	− 0.367	− 0.406	− 0.495	− 0.384	− 0.47	− 0.69	− 0.384	− 0.448	− 0.499
G169S	− 0.217	− 0.268	− 1.502	− 0.409	− 0.354	− 0.621	− 0.359	− 0.289	− 1.507	− 1.461	− 0.314	− 0.517	− 0.386	− 0.191	− 0.518	− 0.37	− 0.251	− 0.585	− 0.349	− 0.241	− 0.481
G169D	− 0.291	− 0.171	− 1.66	− 0.381	− 0.229	− 0.624	− 0.294	− 0.265	− 1.969	− 1.34	− 0.181	− 0.492	− 0.287	− 0.139	− 0.533	− 0.231	− 0.174	− 0.596	− 0.243	− 0.239	− 0.535
A253S	0.175	0.225	0.348	0.067	0.163	0.436	0.106	0.227	0.31	0.402	0.166	0.448	0.08	0.116	0.358	0.139	0.118	0.371	0.161	0.11	0.324
G312A	− 0.061	− 0.068	− 0.09	− 0.057	− 0.037	− 0.049	− 0.046	− 0.09	− 0.101	− 0.101	− 0.068	− 0.083	− 0.051	− 0.048	− 0.05	− 0.075	− 0.089	− 0.067	− 0.07	− 0.092	− 0.097
G331S	− 0.203	− 0.197	− 0.181	− 0.248	− 0.191	− 0.241	− 0.217	− 0.215	− 0.208	− 0.145	− 0.199	− 0.171	− 0.209	− 0.174	− 0.159	− 0.193	− 0.178	− 0.185	− 0.177	− 0.176	− 0.207
G331R	0.033	− 0.024	− 0.008	− 0.003	− 0.003	0.063	− 0.017	− 0.029	− 0.027	− 0.011	− 0.056	0.027	0.004	0.009	− 0.013	− 0.002	− 0.004	− 0.004	0.063	− 0.019	− 0.016

*HK* HDOCK, *HDK* HADDOCK, *CP* ClusPro.

**Figure 4:**
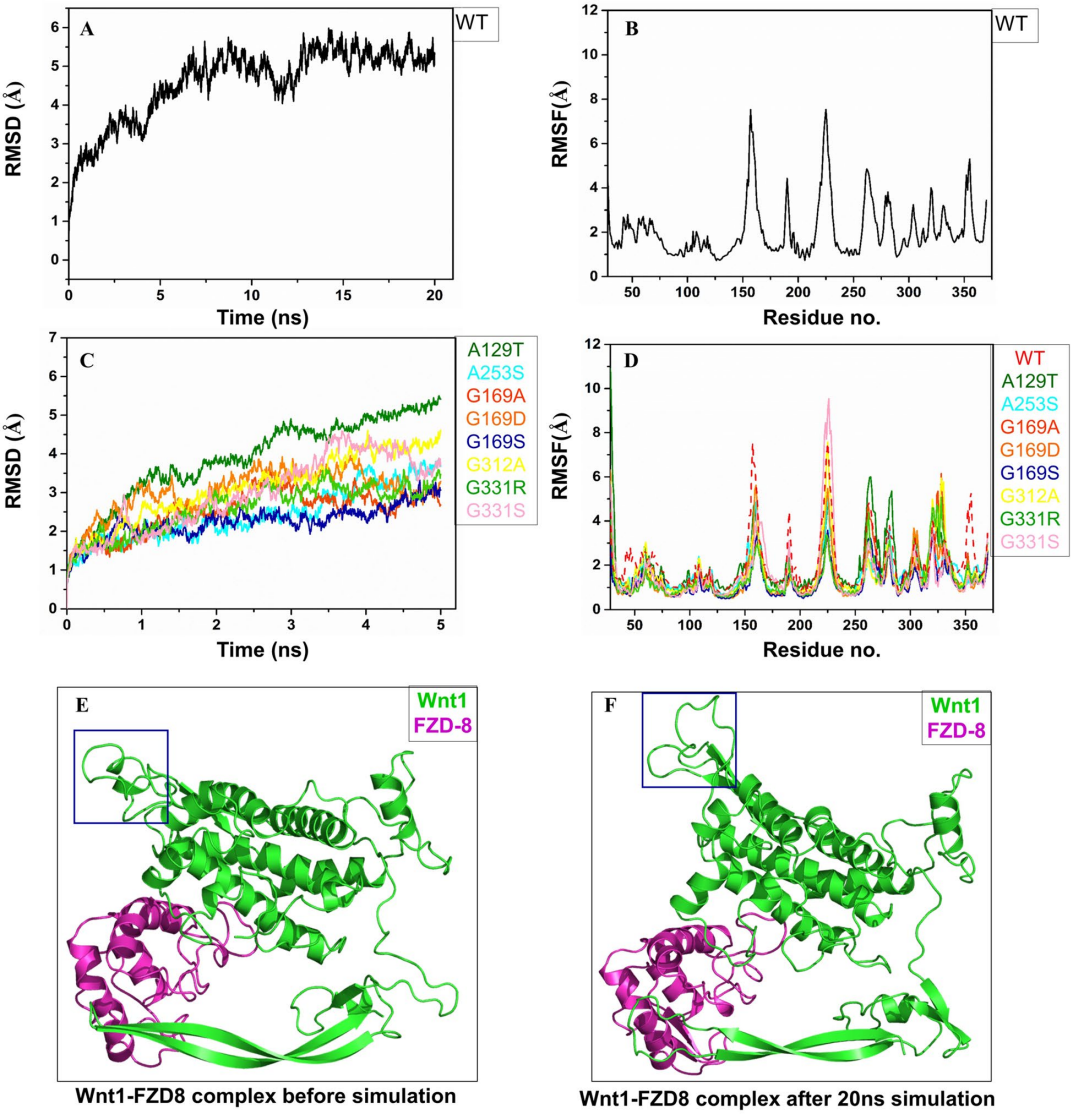
RMSD of Cα atoms of Wnt1 in (**A**) WT and (**C)** Mutated forms in complex states. RMSF of Cα atoms of Wnt1 in (**B**) WT and (**D**) Mutated forms in complex states. (**E**) Modelled structure of Wnt1 before the MD simulation. (**F**) Structure of Wnt1 after the 20 ns MD simulation. Residues 225–245 showing reorientation before and after the MD simulations (marked in blue box) (**E**, **F**).

We found that Wnt1 and FZD8-CRD was the best docked complex in our study, therefore, we performed MD simulation based analyses of wild type Wnt1-FZD8-CRD complex. For complexed WT Wnt1, RMSD increased to 5.5 Å up to ~ 7.5 ns of MD run, and thereafter went on fluctuating in the band of 5 Å ± 1 Å after which there was not much deviation (Fig. 4A). RMSF of WT Wnt1 in complex with FZD8-CRD (Fig. 4B) revealed residue wise fluctuation of the protein. RMSF was higher in the unstructured regions, as expected, especially in the residues 225–245 (Fig. 4E,F). The folded region did not show much deviation from the average structure position, as understood from their lower RMSF values. Among the mutated systems, the A129T showed highest deviation (Fig. 4C), and this might be due to the reorientation of the helix with residues ranging from 260 to 280 (Supplementary Fig. S1). The same was reflected in the RMSF result (Fig. 4D). The G169S mutant system could be considered as most stable because the RMSD and RMSF values were least among all the mutants. Orientations of mutated residues were somewhat different before and after simulation except for the A129T mutant system (Fig. 3).

### Clinical correlation between *Wnt1* expression and deregulation with different cancer types

**Figure 5:**
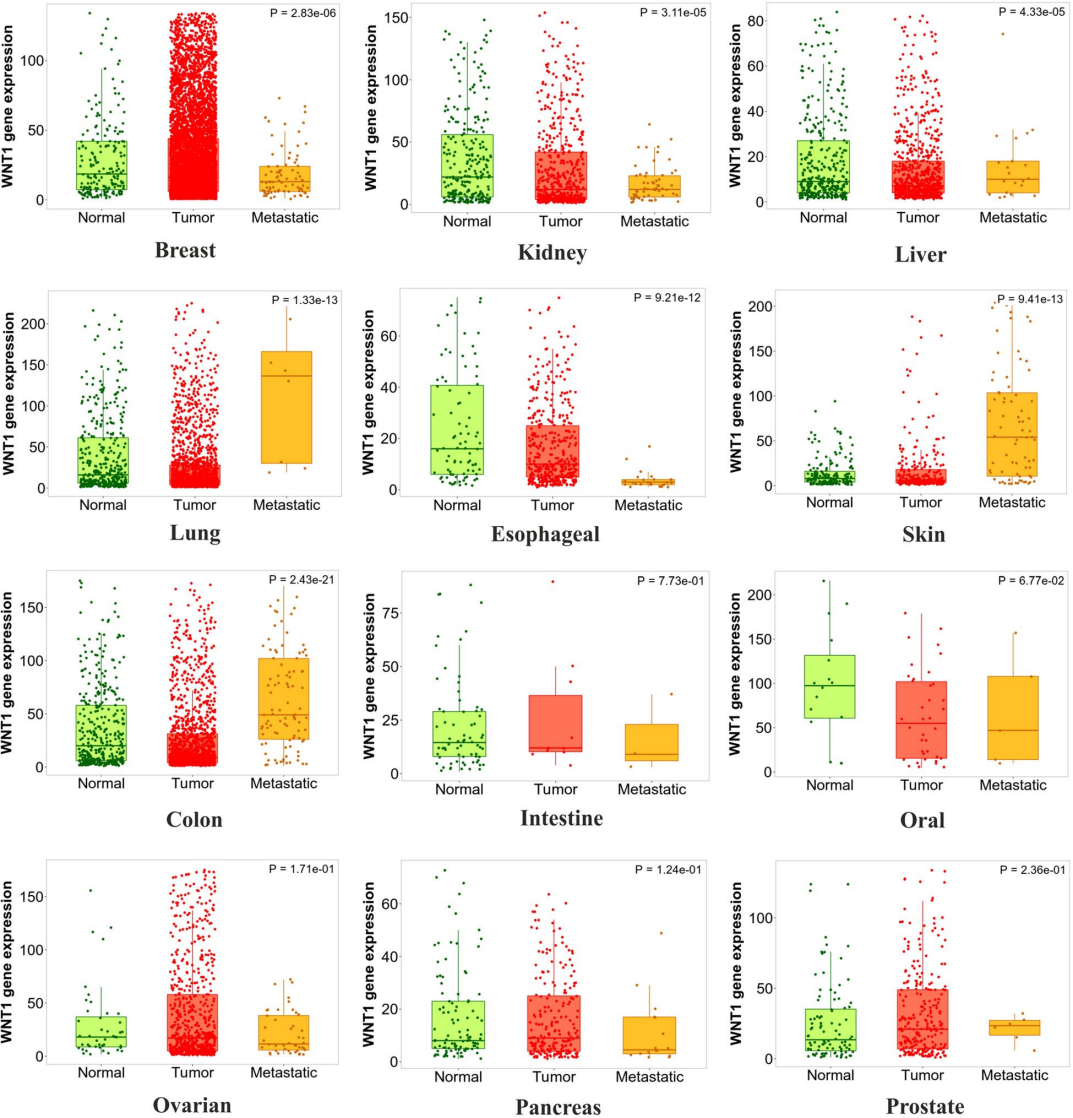
Comparative expression pattern of *Wnt1* gene among various normal, tumor and metastatic tissue samples.

**Figure 6:**
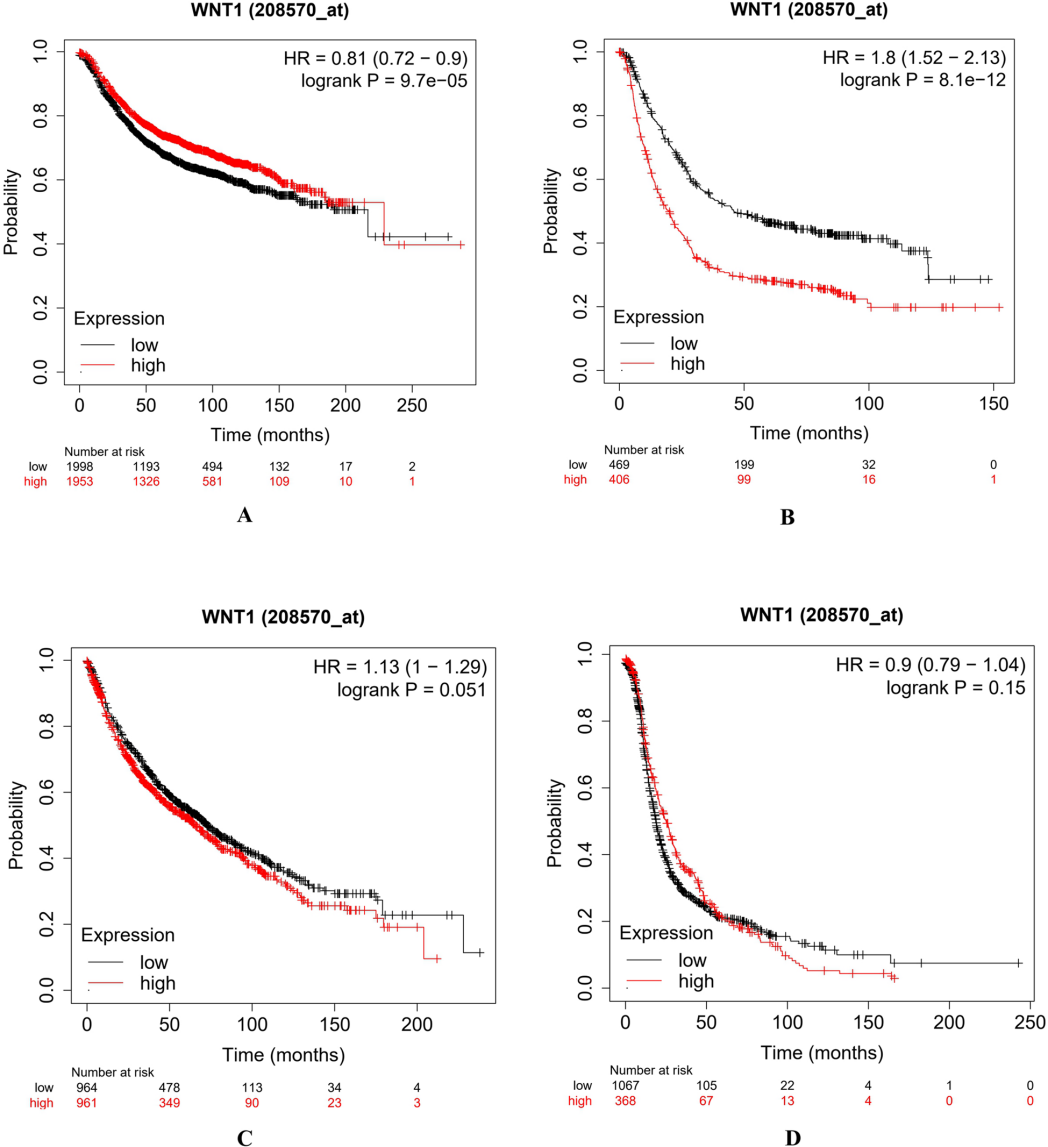
Deregulation of *Wnt1* gene expression and overall survival of patients with breast (**A**), gastric (**B**), lung (**C**) and ovarian (**D**) cancer, using microarray gene expression profile by KM-plotter.

Microarray based differential expression of *Wnt1* gene under Tumor and Normal conditions were carried out in TNM-plot^64^. *Wnt1* mutations were found in different types of cancers like adenomatous polyposis coli, colorectal cancer, and lung cancer, breast cancer, gastric cancer^71^. The *Wnt1* gene expression in different cancer types revealed its importance in tumor progression and cancer formation (Supplementary Fig. S11). We have compared the expression of *Wnt1* gene in normal and tumor samples (Supplementary Table S9) across 21 different tissues (as available in TNM dropdown list). It was observed that *Wnt1* gene was upregulated during tumor formation of endometrium, vulva, ovary and skin; although this upregulated expression was not statistically significant. The *Wnt1* gene expression pattern was then compared among all the twelve normal, tumor and metastatic tissue samples as provided in the TNM dropdown list using gene chip data. It was observed from Fig. 5 and Supplementary Table S10 that *Wnt1* expression was significantly higher (Dunn’s Test p value < 0.01) in metastatic tissues over tumor tissues of lungs, colon and skin; and was significantly lower (Dunn’s Test p value < 0.01) in breast, esophagus and kidney metastatic tissues over tumor tissues. As deregulation of Wnt1 activity was associated with various cancers and other human diseases^8,9^, a meta-analysis was also carried out to assess the overall survival rate of breast, gastric, lungs and ovarian cancer patients with *Wnt1* expression, using Kaplan–Meier plotter (Fig. 6); and we found a strong relation between Wnt1 deregulation and the overall survival rate^72^. In case of breast cancer patients, Kaplan‐Meier curve and log‐rank test analyses showed that high expression of *Wnt1* (HR = 0.83; P = 0.0004) was associated with less number of patients at risk. This observation also correlates with the data obtained from TNM-plot which shows that *Wnt1* has significantly lower expression in metastatic breast tissues. In case of gastric cancer (HR = 1.8; P > 0.00001) and lung cancer (HR = 1.13; P = 0.051) patients, higher expression of *Wnt1* was associated with less survival rate (i.e. more patients at risk)^73^. From TNM-plot we have also observed significantly higher expression of *Wnt1* in metastatic tissues of lung. On the other hand, in the case of ovarian cancer patients, *Wnt1* expression (HR = 0.9; P = 0.15) was thought to have no such effects.

## Discussion

In our present study, we have addressed the structural and functional implications of a large number of nsSNPs of human *Wnt1* gene using multiple sequence and structure based approaches. Use of multiple softwares and algorithms increase the confidence during prediction of the effect of missense mutations on a protein^67^.

Initial screening of 292 nsSNPs of *Wnt1* using multiple sequence based algorithms prioritized 10 highly deleterious nsSNPs (A129T, G169S, G169D, G169A, C218Y, A253S, G312A, G331S, G331R, W351G). As random coil is the major contributor of Wnt1 structure, and a considerable number of deleterious nsSNPs are present within this random coil region of protein, therefore, these nsSNPs may impact on flexibility of Wnt1 protein^67^. Amino acid substitutions at the highly conserved regions, often involved in important biological functions, tend to be more deleterious in nature than nsSNPs which are located at less conversed sites^74,75^. Consurf analyses revealed that 46.23% of total nsSNPs occurred in the conserved areas of the protein. It was found from Consurf analyses that the positions for all the 10 highly deleterious nsSNPs were highly conserved. ConSurf predicted that the variant A129T and C218Y occurred at important structural residues (highly conserved and buried) whereas, G169A, G169D, G169S, G312, G331R and G331S occurred at important functional residues (highly conserved and exposed).

Being a multi-functional protein, Wnt1 interacts with numerous partners at the membrane as well as in the cytosol^76^. Protein–protein interaction (PPI) networks are important to understand the functional impact of the protein and its interacting partners^77^. Therefore, amino acid substitutions in Wnt1 due to nsSNPs, may alter the binding interfaces in the PPI network, which may have crucial importance in Wnt1 secretion, transport, canonical and non-canonical Wnt signaling. Residues 214–234 of Wnt1 are involved in porcupine (PORCN)-dependent palmitoylation^52^, which is required for its secretion through binding of palmitoylated Wnt1 to Wntless (WLS)^78^. Therefore, nsSNPs occurring in this region (like M214I, R215G, R215L, E217K, C218G, C218Y, H221Y, G225S, S226L, C227G, C227Y, V229A, R230H) of *Wnt1* may potentially alter the interacting interface between Wnt1 and porcupine and thereby may hinder its secretion. SIFT had predicted all these variants as deleterious and REVEL had predicted these variants as likely disease causing. Interestingly, variant like C218Y, occurred within this PTM sequence site of Wnt1 also present within the 10 highly deleterious nsSNPs. Double acylation (O-acylation at position C93 and S-acylation at position S224) in Wnt1 was essential for its secretion as well as for its activity. Studies showed that Wnt1 mutants C93A and S224A were trapped in the ER, impairing their secretion^75^. The local structure around the palmitoylation site is important for recognition of Wnt1 substrates by PORCN. Therefore, structural alterations in these regions due to nsSNPs might affect the palmitoylation of Wnt1 by PORCN^79^. Variants A129T and G169D are located within the N-terminal domain, and form hydrogen bonds with amino acid position at 120, thus making the structure unstable^5^. Variant A129T also changes the binding cavity around the mutant residues^9^ and increases the relative solvent accessibility. Upon amino acid substitutions in Wnt1, the major structural and post-translational modifications were gain of relative solvent accessibility, altered disordered interface, altered ordered interface, altered transmembrane protein, loss and gain of catalytic sites, loss and gain of disulphide linkage, gain and loss of allosteric sites, altered metal binding, gain of intrinsic disorder, gain and loss of strand, altered stability, loss of phosphorylation, loss and gain of N-linked glycosylation.

It is well known that the function of a protein is directly depends on its tertiary structure^80^. Therefore, the substitution of amino acids due to nsSNPs may impact on structural conformation of Wnt1 that may alter its potential physiological functions. Majority of the disease associated nsSNPs affected the stability of the protein^81^ and it was found that 8 out of 10 highly deleterious variants of Wnt1 showed strong destabilizing effects by all the five prediction methods. Furthermore, variants like G169S, G169D, G169A, A253S, G312A, G331S and G331R are located within the loop region of Wnt1 structure, which may affect the flexibility of the protein. Further investigation of the structural deviation of the 8 high risk destabilizing variants of Wnt1 revealed that G169S and G331S showed higher RMSD values from wild type; whereas, G331R showed lowest RMSD value^74^. Therefore, the structural variation of Wnt1 at local or global scale may impair its interaction with the partner proteins in the network^79^. As the protein dynamics play an important role in molecular recognition as well as in catalytic activity and as the mobility of a protein is an intrinsic property, encrypted in its primary structure, we have performed NMA study to examine whether Wnt1 and its 8 highly destabilizing variants display any unique patterns of intrinsic mobility^82^. Based on the deformation energies obtained from the normal modes, it was observed that some variants became unstable. Further insights from MD simulations of WT Wnt1 revealed the close state of the binding pocket of Wnt1 for CRDregions of frizzled receptors in absence of ligands. It was reported that Klotho-derived peptides facilitated the close to open state transition of FZD-CRD binding pocket of Wnt-1^52^. Therefore, this structural transition might be necessary for Wnt1- FZD-CRD interaction. It was also reported that Wnt4 showed higher fluctuation rate in residues nearer to edges of two domains, the thumb (NTD) and index finger (CTD), involved in FZD-CRD interactions^83^. The unstructured loop regions of Wnt1 showed fluctuations, revealing the flexibility of the protein^84^. Interestingly, RMSF values in the binding pocket for FZD-CRDs (residue 330–360) were lower compared to other regions of the Wnt1. RMSD and RMSF may also account for the atomic fluctuations due to changes in molecular orientations of a protein around their average conformations and serve as important indicators of dynamic behavior of several biological processes^83^. The superimposition of WT Wnt1 and its 8 prioritized variants before and after simulation clearly showed positional shifts of side chain structures. The exact mechanism and the role of those predicted nsSNPs, which influenced the Wnt1 stability, should be validated experimentally. Wnt1 binds at the CRD regions of frizzled receptors- FZD-1, FZD-2, FZD- 4, FZD-5, FZD-7, FZD-8 and FZD-10^52^. Previous studies reported that, proteins-protein interaction were mediated through their interfaces that generally accomplishing their functions^85^. In addition, the residue properties, involved in these interfaces, also determine the binding specificity and affinity^86^. In this context, we further intended to investigate the effects of highly destabilizing variants of Wnt1 on the binding interface and to explore the receptor selectivity towards Wnt1 and the downstream signaling cascade. Among 8 highly deleterious variants, G169D highly destabilize the binding interfaces of all 7 Wnt1-FZD-CRD complexes specially the Wnt1-FZD-5-CRD complex. Since about 6 binding amino acids (viz., L47, R91, W95, R240, N346 and F349) of Wnt1 are most commonly shared with all the Wnt1-FZD-CRDs complexes of our study, it can be inferred that all 7 FZD-CRDs bind in the similar region of Wnt1. Further analyses of Wnt1 nsSNPs, present in the Wnt1-FZD-CRDs interacting interface, revealed that several amino acid substitutions had destabilizing effects on the interface. Whereas, variants like H221Y, T348I, H362Q, and Q351C may increase the downstream signaling cascade by stabilizing the complexes of Wnt1 with CRDs of FZD5, FZD4, FZD2 and FZD7, respectively. So, these variants may affect and even disrupt interactions of the mutated Wnt1 protein in a particular PPI network^86^. This could affect some of the interactions of the Wnt1 protein or "edges" in canonical Wnt signaling, which could have functional consequences^87^. Another important variant of Wnt1, R235W disrupts the structure of Wnt1 thumb region by destabilizing the β-strand that supports FZD-CRD binding loop. The Trp side chain in the R235W variant would clash with the side chains of Trp233, Arg141, and Asp172 and may seem to alter the interaction with FZD receptor^88^. Furthermore, Cys227, Leu239, Arg240 and Glu343 residues are involved in binding with FZD-CRDs^52^, therefore substitutions in these positions (C227G, C227Y, L239P, R240C and E343Q) may disrupt the Wnt1- FZD-CRDs binding interface. Further experimental works are needed to validate the destabilizing effects of Wnt1 variants in the binding interface. As Wnt1-FZD8-CRD was the best docked complex, therefore, analyses of Wnt1-FZD8-CRD interaction through MD simulation revealed that RMSF values were decreased in the index (CTD) and thumb (NTD) region of Wnt1, indicating strong interactions between them^84^. There is a hint that mutations in Wnt1 perturb the intra-molecular interactions of the protein compared to the same in WT, and therefore it seems that the mutations might have functional consequences on the protein.

The genes exhibit comparable expression profiles between cancerous and normal tissues, can serve as either targets of treatment or molecular biomarkers of cancer progression^8^. Comparing the expression of *Wnt1* gene in normal and tumor samples across 21 different tissues had revealed no statistically significant up or down regulation of this gene. Further, when the *Wnt1* gene expression pattern was compared among normal, tumor and metastatic tissue samples, it was observed that *Wnt1* expression was significantly higher in metastatic tissues of lungs, colon and skin^71,89,90^. KM Plot analyses revealed that Wnt1 deregulation affected overall survival in gastric and breast cancer patients and it had no impact on the survival rate of lung and ovarian cancer patients. This observation may be due to functional redundancy of both Wnt1 and FZD receptors, and thereby suggests differential regulation of subsequent downstream signaling cascade^76^. In case of breast cancer, high expression of *Wnt1* was associated with less number of patients at risk. This inverse relationship between *Wnt1* expression pattern and overall survival rate of breast cancer patients may be due to oncogenic regulation of FZD1, one of the Wnt1 binding receptor^91^. In breast cancer, nestin, an extracellular matrix (ECM) intermediate filament protein, inhibits FZD1 expression and thereby β-catenin signaling, resulting the halt of proliferation and invasion of breast tissues by decreasing the expression of matrix metallopeptidase^92^. Therefore, *Wnt1* differential gene expression pattern may be used as a prognostic biomarker to predict overall survival of the gastric and breast cancer patients. As nsSNPs may deregulate the encoded protein^80^, the structurally destabilizing variants identified in our study, may have similar functional consequences in Wnt1 deregulation.

In conclusion, our results demonstrate that several nsSNPs in the *Wnt1* gene may be deleterious to its structure and functions. We have identified 10 highly deleterious and destabilizing nsSNPs of *Wnt1*. We found that several nsSNPs affect Wnt1 post-translational modifications, important for its protein–protein interaction and signaling. Thirteen nsSNPs of *Wnt1* may potentially alter the interaction interface between Wnt1 and porcupine and thereby impact on its secretion. We have identified highly destabilizing variants of Wnt1 on the binding interface with FZD-CRDs, which may alter the downstream Wnt1 signaling cascade. MD simulation of apo Wnt1 and complex of Wnt1-FZD8-CRD revealed the open and close state transition of Wnt1 upon interaction with FZD-CRDs. Furthermore, MD simulation also showed that amino acid substitutions in Wnt1 might perturb the intra-molecular interactions of the protein compared to the same in wild type. *Wnt1* showed differential expression profiles between normal, tumor and metastatic tissues and its deregulation affected survival outcome in patients with lung and gastric cancer. Although bioinformatics tools have their own limitations, our present computational study may be useful in further population based researches and towards development of precision medicines for the treatment of diseases caused by the most deleterious nsSNPs of *Wnt1*. Further experimental works are required to elucidate the deleterious nature of the prioritized nsSNPs of *Wnt1*.

## Supplementary Information

Supplementary Legends.

Supplementary Figure S1.

Supplementary Figure S2.

Supplementary Figure S3.

Supplementary Figure S4.

Supplementary Figure S5.

Supplementary Figure S6.

Supplementary Figure S7.

Supplementary Figure S8.

Supplementary Figure S9.

Supplementary Figure S10.

Supplementary Figure S11.

Supplementary Figure S12.

Supplementary Table S1.

Supplementary Table S2.

Supplementary Table S3.

Supplementary Table S4.

Supplementary Table S5.

Supplementary Table S6.

Supplementary Table S7.

Supplementary Table S8.

Supplementary Table S9.

Supplementary Table S10.

## Data Availability

The datasets generated and/or analyzed during the current study are available in the Ensembl genome browser and UniProt repository, (https://asia.ensembl.org/Homo_sapiens/Gene/Variation_Gene/Table?db=core;g=ENSG00000125084;r=12:48978322-48982620; https://www.uniprot.org/uniprot/P04628).
