# Supplementary material for: In silico analyses of Wnt1 nsSNPs reveal structurally destabilizing variants, altered interactions with Frizzled receptors and its deregulation in tumorigenesis
Source: Sci Rep. 2022 Sep 2;12:14934. doi: 10.1038/s41598-022-19299-x (PMC9440047; doi:10.1038/s41598-022-19299-x)
Supplement: Supplementary file 1 — Supplementary Legends. [file 41598_2022_19299_MOESM1_ESM.docx]

**Supplementary Table and Figure Legends**

**Supplementary Table S1.** Data of nsSNPs from Ensemble variation table (Supplementary Table S1. Data of nsSNP from Ensemble variation table (Assembly: GRCh38, Accessed on 14.10.2020; URL: https://asia.ensembl.org/Homo_sapiens/Gene/Variation_Gene/Table?db=core;g=ENSG00000125084;r=12:48978322-48982620).

**Supplementary Table S2.** Initial screening of 292 nsSNPs of human *Wnt1* gene using multiple web-servers.

**Supplementary Table S3.** Stability prediction by I-Mutant 3.0 server.

**Supplementary Table S4.** List of conservation score wise amino acid distribution in human Wnt1 by ConSurf server.

**Supplementary Table S5.** Effect of 10 nsSNPs on the structure, functions and post-translational modifications of human Wnt1 as predicted by MutPred2 web-server.

**Supplementary Table S6.** Residues interaction pattern in the binding interface of wild type Wnt1 and seven FZD-CRD complexes obtained from HDOCK, HADDOCK and ClusPro servers by PDBsum.

**Supplementary Table S7.** Stability prediction of binding interfaces between all variants coincide with positions of nsSNPs present in Wnt1 binding interface and seven FZD-CRDs by mCSM.

**Supplementary Table S8.** *Wnt1* gene expression with corresponding fold changes in 21 different cancer types by TNM-plot.

**Supplementary Table S9.** Wnt1 gene expression pattern in twelve normal, tumor and metastatic tissue samples with their corresponding tumor-metastatic fold changes by TNM-plot.

**Supplementary Figure S1**. Flexibility plot of amino acids in human Wnt1.

**Supplementary Figure S2.**  Disorder regions and globular domains of human wild type Wnt1 and 10 Wnt1 variants by GlobPlot 2.3.

**Supplementary Figure S3.**  Secondary structure of human wild type Wnt1 and 10 Wnt1 variants by PSIPRED.

**Supplementary Figure** **S4.** Multiple sequence alignment of human wild type Wnt1 and its 10 variants by Clustal Omega.

**Supplementary Figure** **S5.** Protein-protein interaction patterns of human Wnt1.

**Supplementary Figure** **S6.** Pictures and Ramachandran plots of human Wnt1, modeled from different modeling servers and PDBsum respectively.

**Supplementary Figure** **S7.** 10 Wnt1 variants modeled by I-TASSER.

**Supplementary Figure** **S8.** Graphical representation of BC score, RIMSIP score and deformation energy by Webnma.

**Supplementary Figure** **S9.** Interacting interfaces between human wild type Wnt1 and CRD regions of different Frizzled receptors by PDBsum.

**Supplementary Figure** **S10A.** 2D plot of human wild type Wnt1 vs different FZD-CRD complexes obtained from HDOCK server by LigPlot+v.2.2.5

**Supplementary Figure** **S10A.** 2D plot of human wild type Wnt1 vs different FZD-CRD complexes obtained from HADDOCK server by LigPlot+v.2.2.5

**Supplementary Figure** **S10A.** 2D plot of human wild type Wnt1 vs different FZD-CRD complexes obtained from ClusPro server by LigPlot+v.2.2.5

**Supplementary Figure S11.**  Pan-cancer analyses by TNMplot. Boxplot of *Wnt1* gene differentially expressed in different tumor types. Significant differences by a Mann–Whitney U test are marked with red color (* p < 0.01).

**Supplementary Figure S12.** Flowchart depicting the detailed procedure of analyses in TNMplot (A) and in Kalpan-Meier plotter (B).
