# Supplementary figures and images for: In silico analyses of Wnt1 nsSNPs reveal structurally destabilizing variants, altered interactions with Frizzled receptors and its deregulation in tumorigenesis
Source: Sci Rep. 2022 Sep 2;12:14934. doi: 10.1038/s41598-022-19299-x (PMC9440047; doi:10.1038/s41598-022-19299-x)

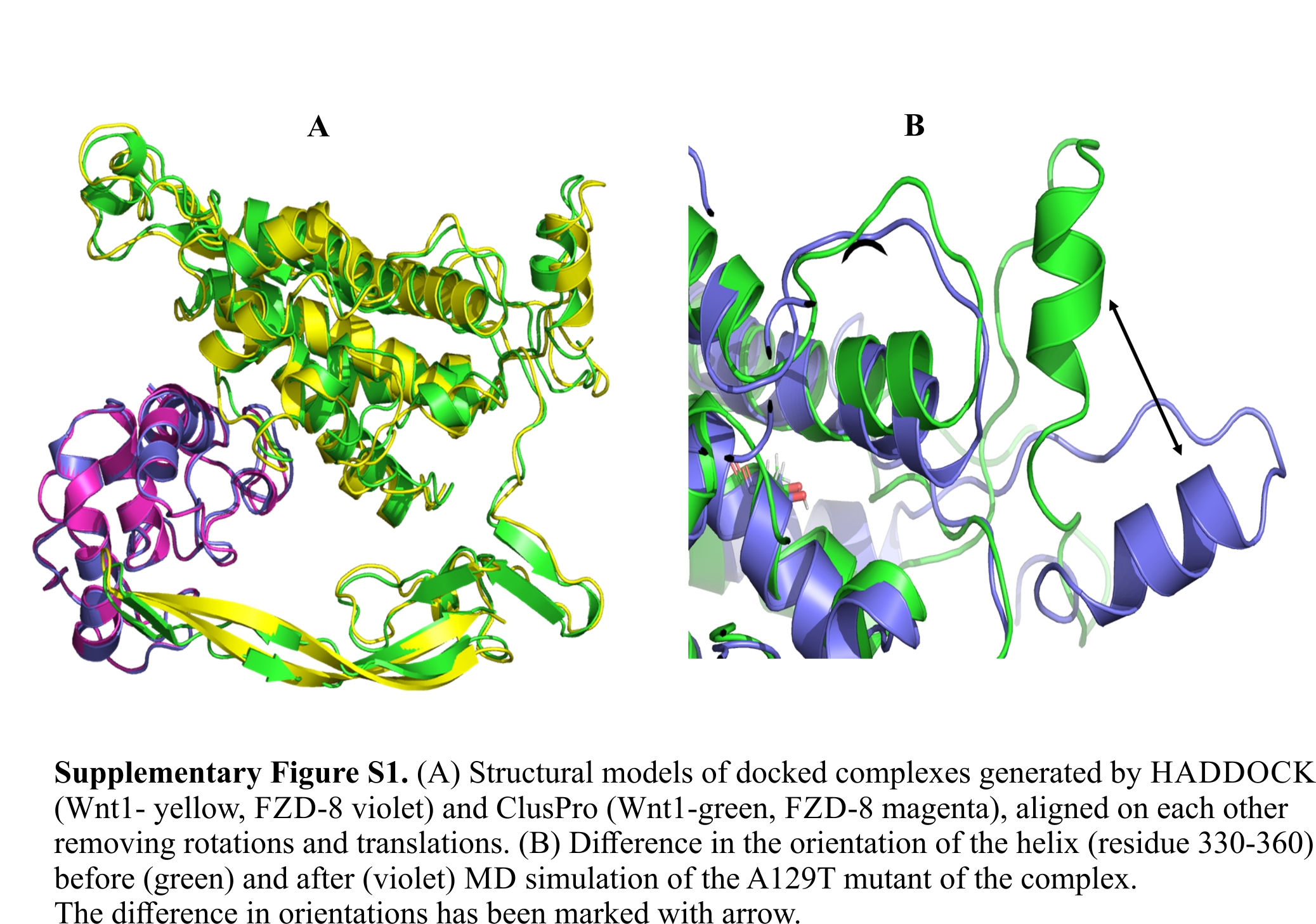

Supplement: Supplementary file 2 — Supplementary Figure S1. [file 41598_2022_19299_MOESM2_ESM.jpg]

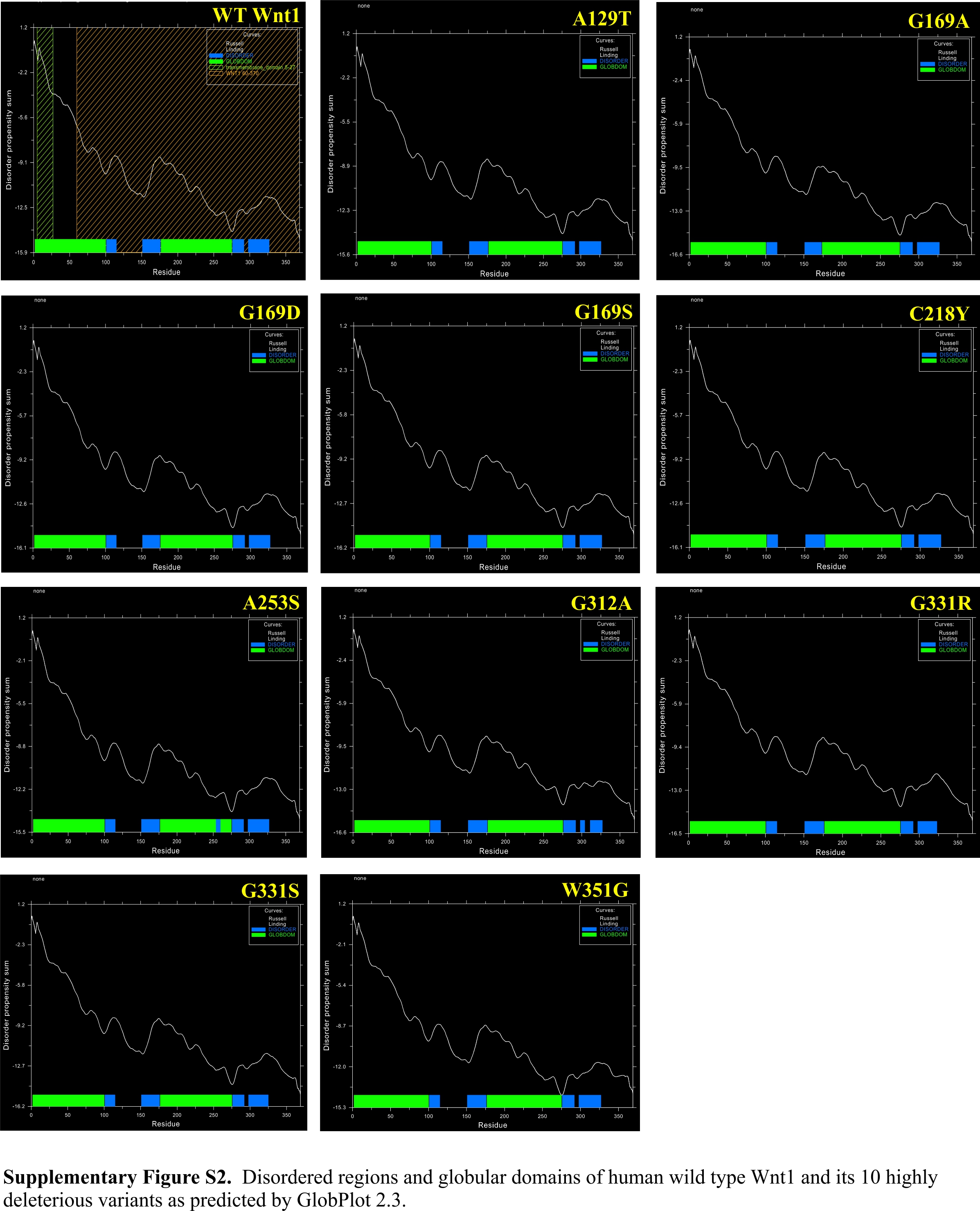

Supplement: Supplementary file 3 — Supplementary Figure S2. [file 41598_2022_19299_MOESM3_ESM.jpg]

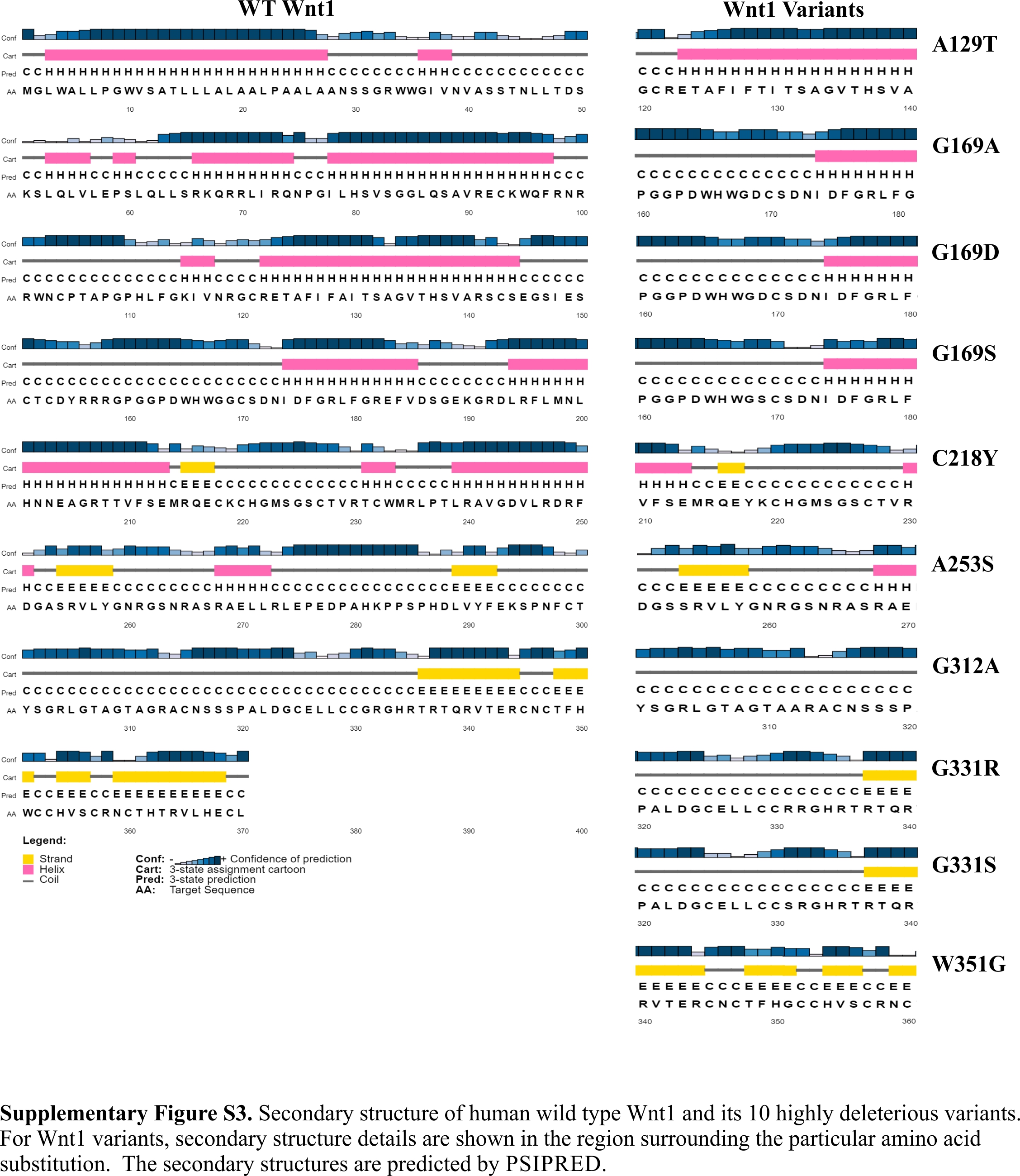

Supplement: Supplementary file 4 — Supplementary Figure S3. [file 41598_2022_19299_MOESM4_ESM.jpg]

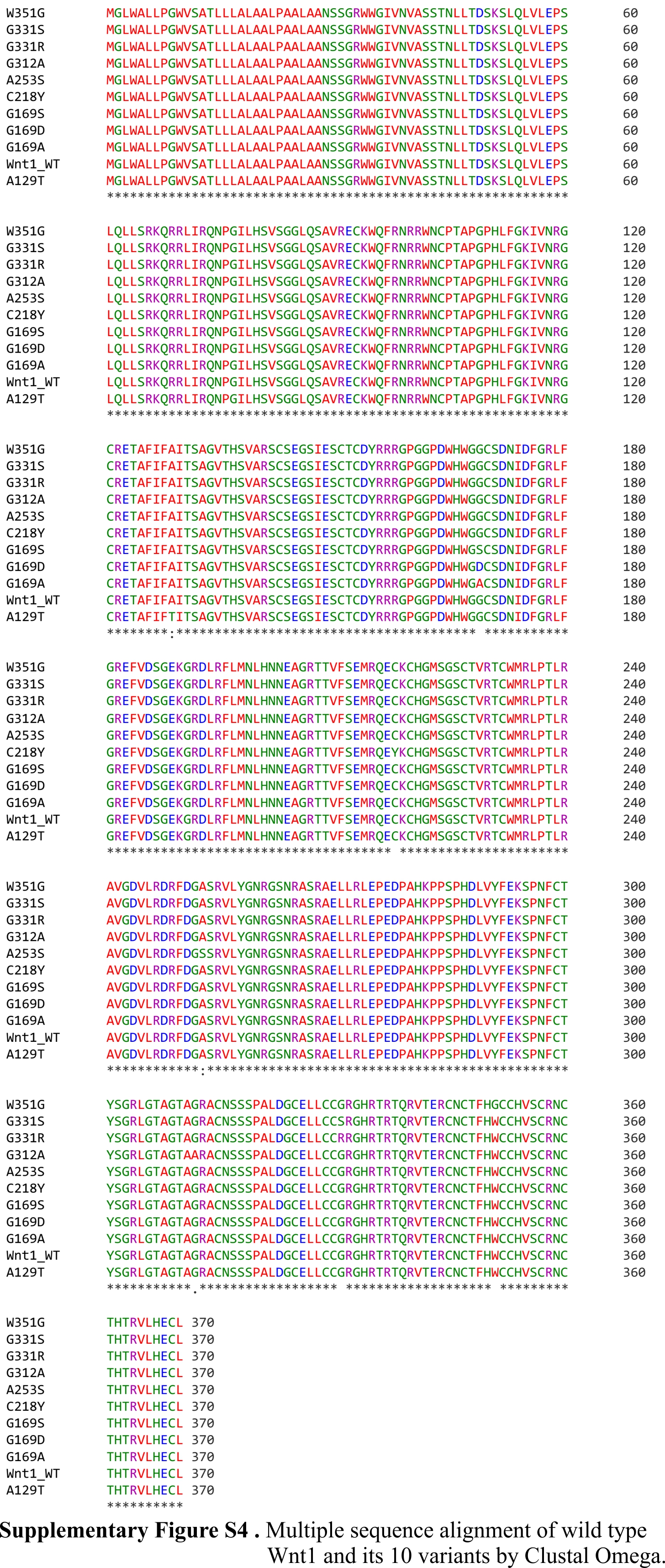

Supplement: Supplementary file 5 — Supplementary Figure S4. [file 41598_2022_19299_MOESM5_ESM.jpg]

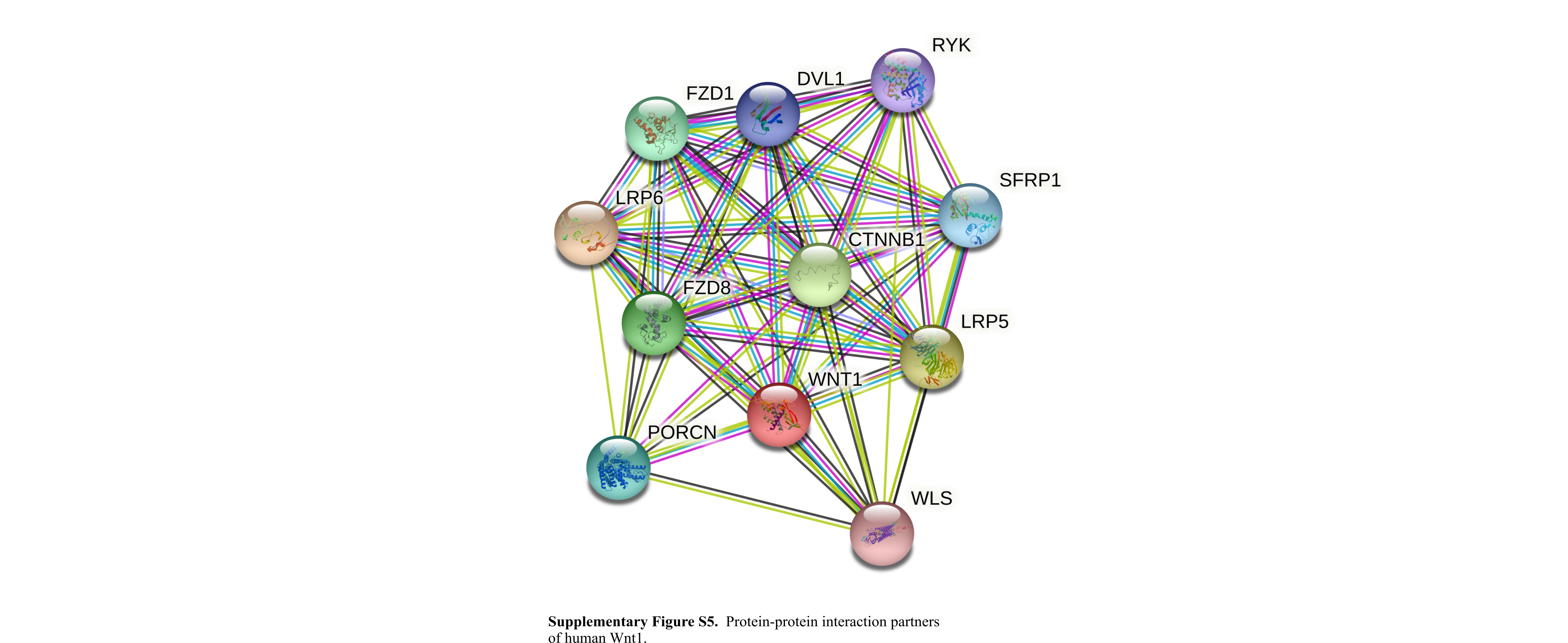

Supplement: Supplementary file 6 — Supplementary Figure S5. [file 41598_2022_19299_MOESM6_ESM.jpg]

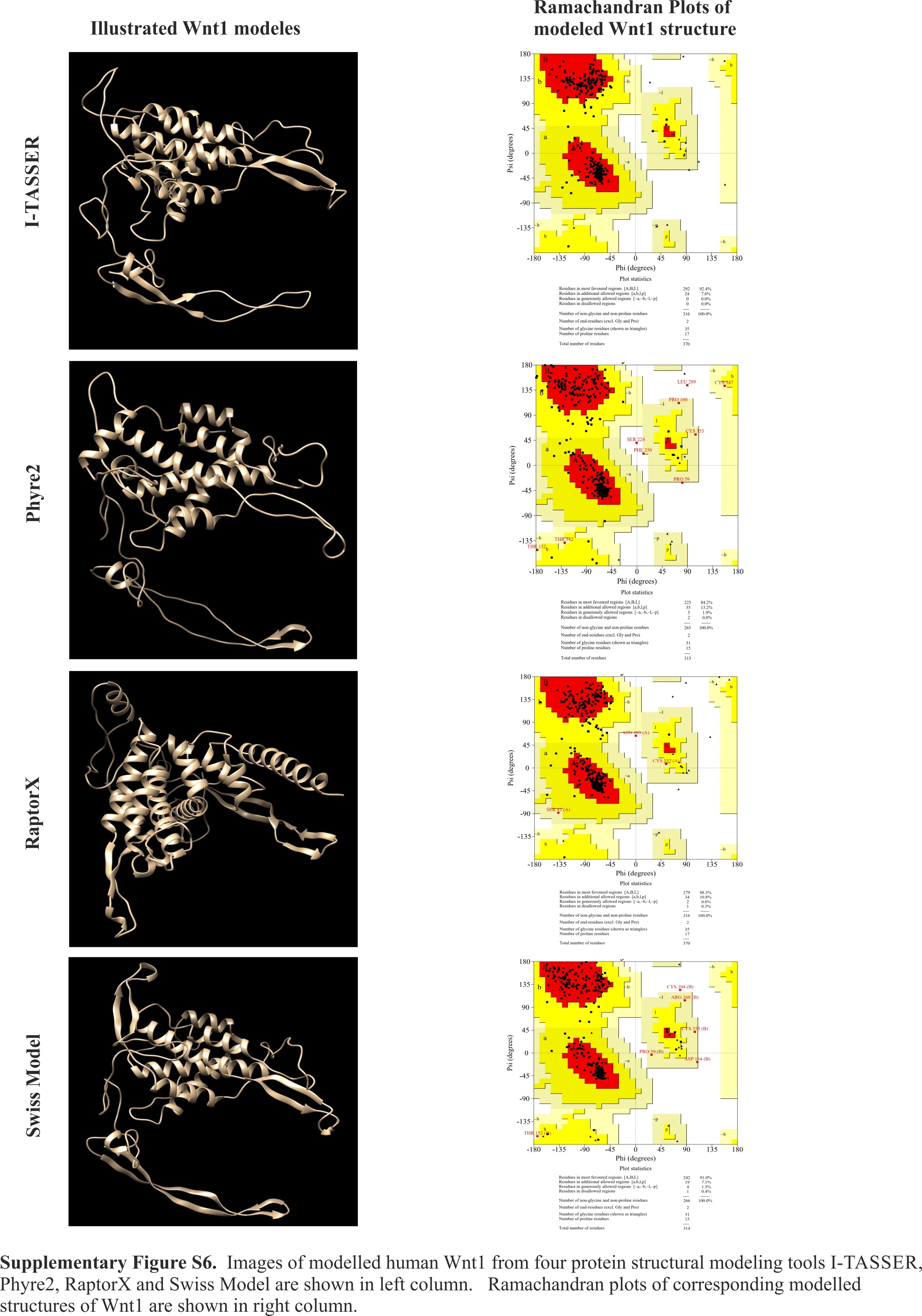

Supplement: Supplementary file 7 — Supplementary Figure S6. [file 41598_2022_19299_MOESM7_ESM.jpg]

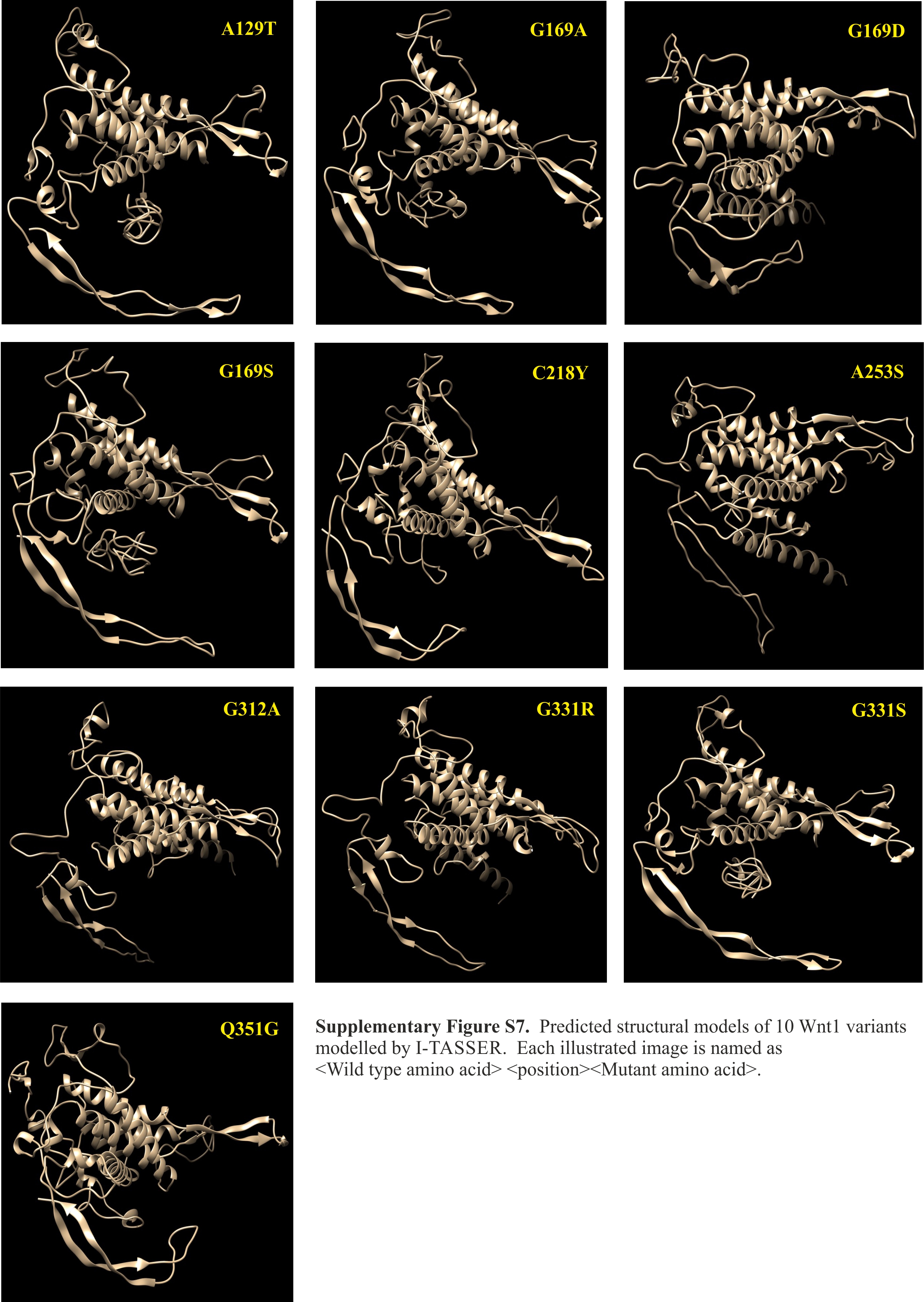

Supplement: Supplementary file 8 — Supplementary Figure S7. [file 41598_2022_19299_MOESM8_ESM.jpg]

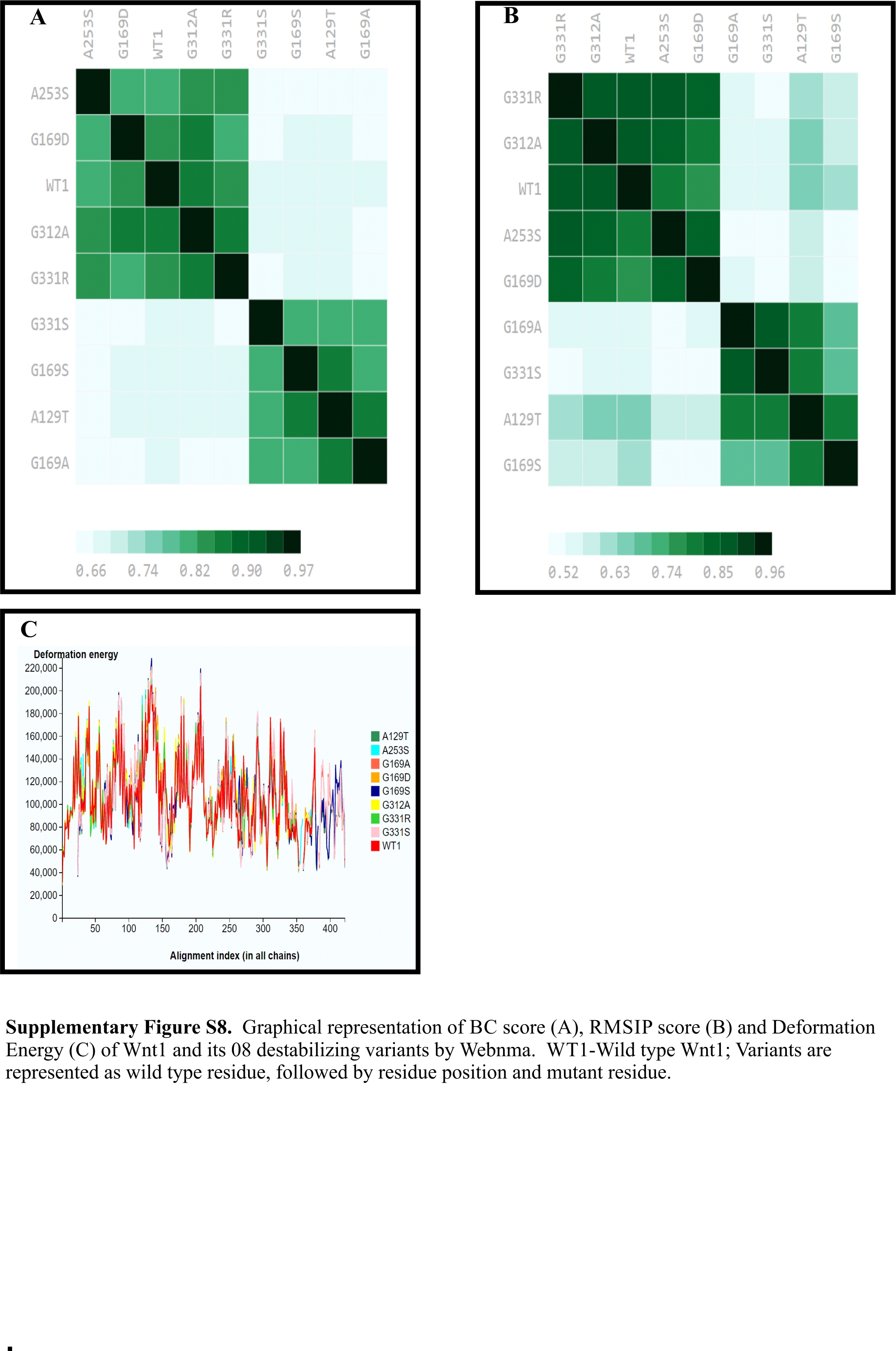

Supplement: Supplementary file 9 — Supplementary Figure S8. [file 41598_2022_19299_MOESM9_ESM.jpg]

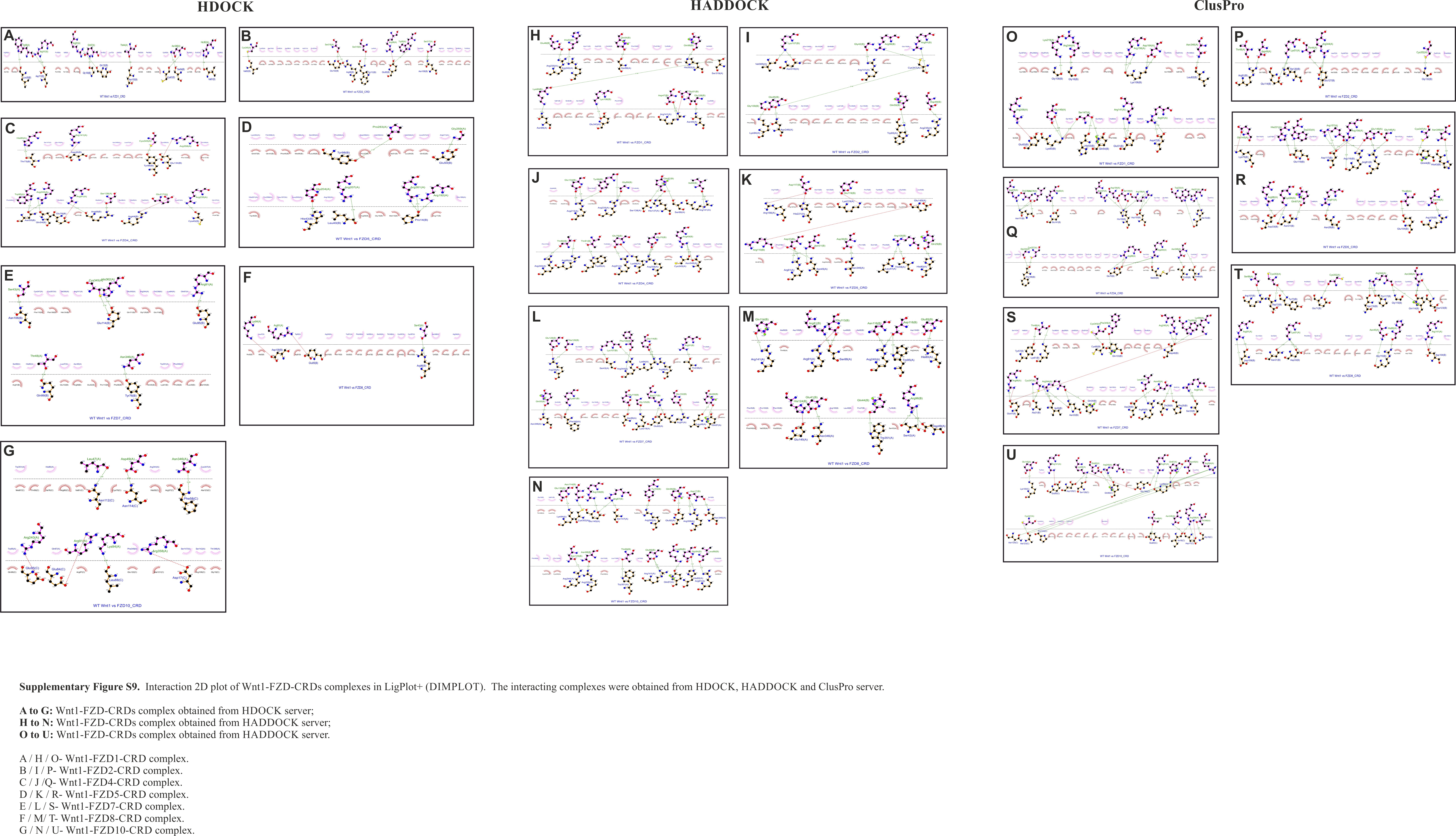

Supplement: Supplementary file 10 — Supplementary Figure S9. [file 41598_2022_19299_MOESM10_ESM.jpg]

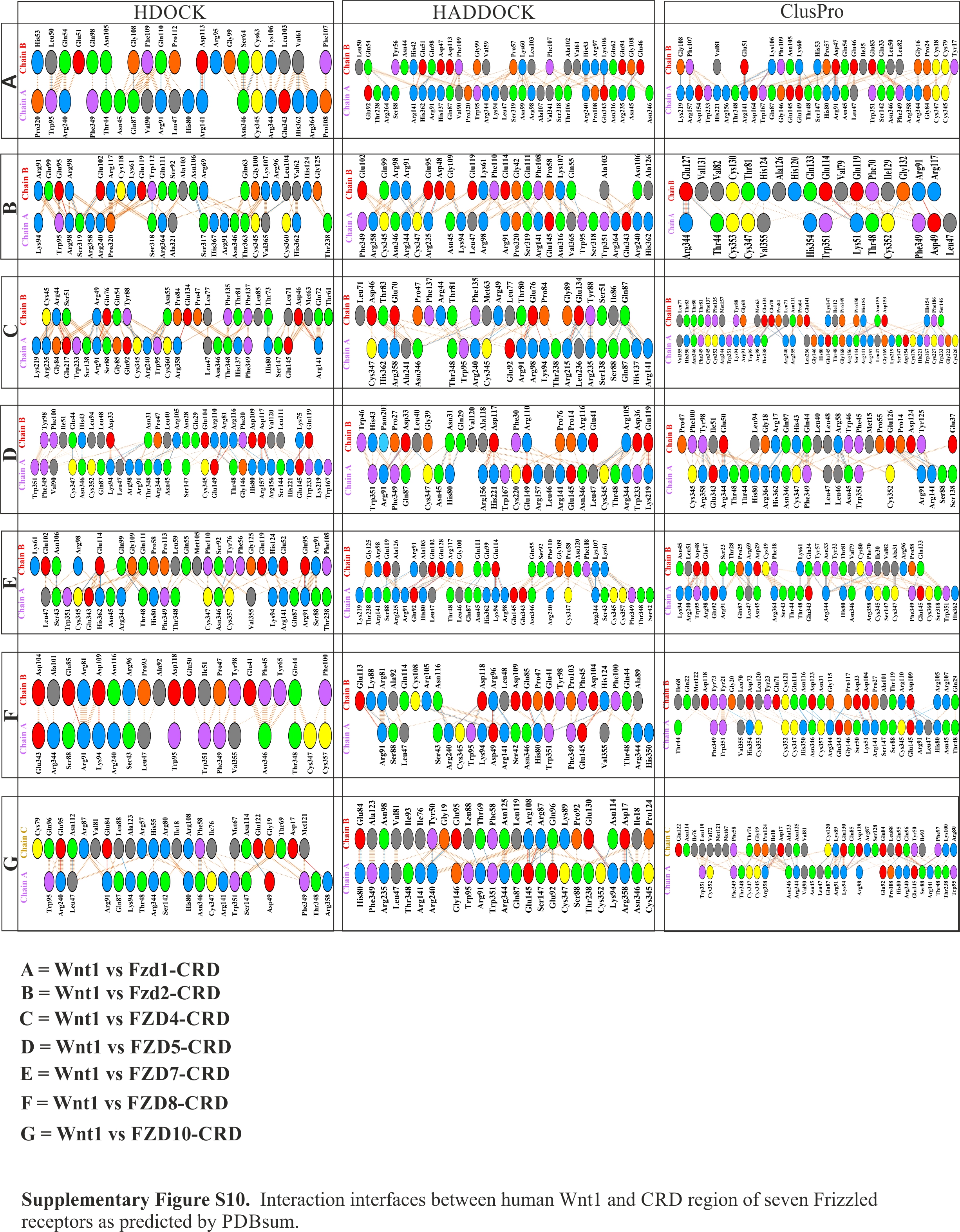

Supplement: Supplementary file 11 — Supplementary Figure S10. [file 41598_2022_19299_MOESM11_ESM.jpg]

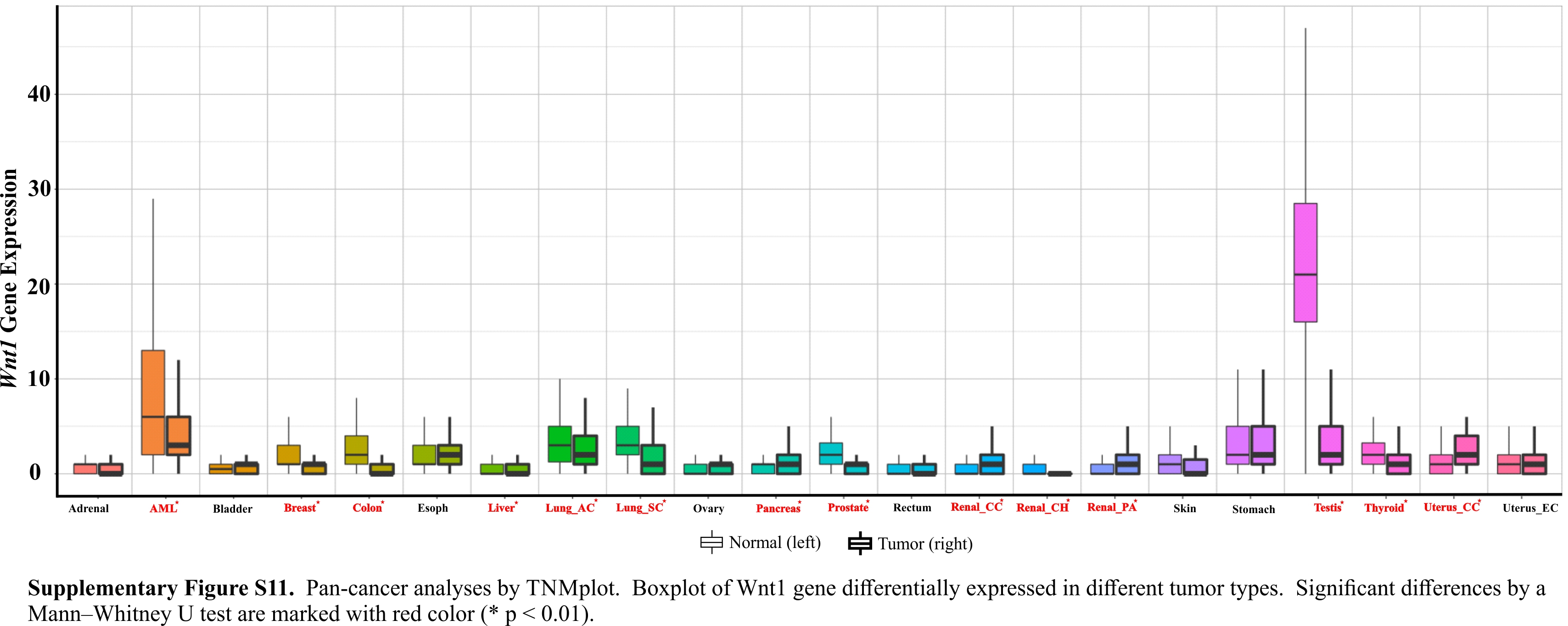

Supplement: Supplementary file 12 — Supplementary Figure S11. [file 41598_2022_19299_MOESM12_ESM.jpg]

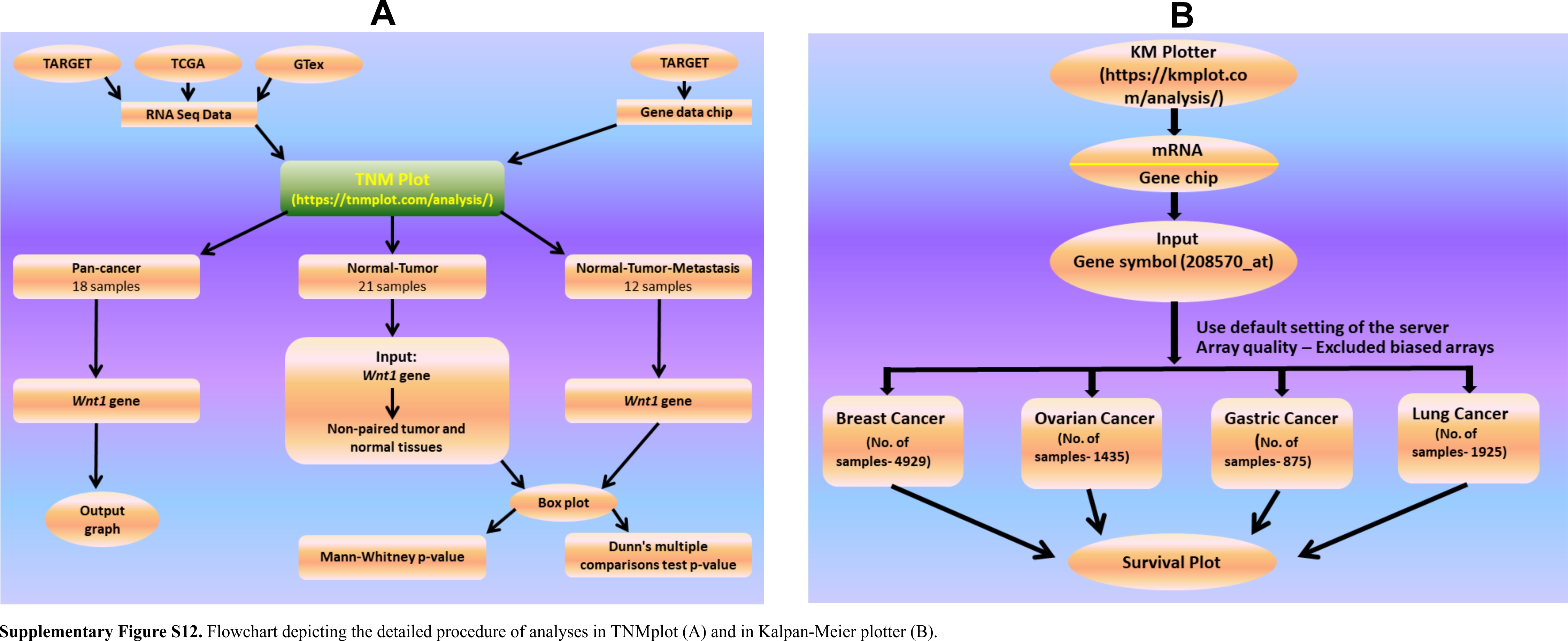

Supplement: Supplementary file 13 — Supplementary Figure S12. [file 41598_2022_19299_MOESM13_ESM.jpg]
