# Supplementary material for: In silico analyses of Wnt1 nsSNPs reveal structurally destabilizing variants, altered interactions with Frizzled receptors and its deregulation in tumorigenesis
Source: Sci Rep. 2022 Sep 2;12:14934. doi: 10.1038/s41598-022-19299-x (PMC9440047; doi:10.1038/s41598-022-19299-x)
Supplement: Supplementary file 14 — Supplementary Table S1. [file 41598_2022_19299_MOESM14_ESM.docx]

Supplementary Table S1: List of simulated Wnt1 without and with FZD-8.

| Name of the system | | Residue no. | Simulation length |
| --- | --- | --- | --- |
| Wnt1 without FZD-8  (Apo Wnt1) | WT | 28-370 | 20ns |
|  | A129T |  | 5ns |
|  | A253S |  | 5ns |
|  | G169A |  | 5ns |
|  | G169D |  | 5ns |
|  | G169S |  | 5ns |
|  | G312A |  | 5ns |
|  | G331R |  | 5ns |
|  | G331S |  | 5ns |
| Wnt1 complexed with FZD-8 | WT | 28-370 (Wnt1)  6-124 (FZD-8) | 20ns |
|  | A129T |  | 5ns |
|  | A253S |  | 5ns |
|  | G169A |  | 5ns |
|  | G169D |  | 5ns |
|  | G169S |  | 5ns |
|  | G312A |  | 5ns |
|  | G331R |  | 5ns |
|  | G331S |  | 5ns |
